# Impaired network organization in mild age‐related hearing loss

**DOI:** 10.1002/mco2.70002

**Published:** 2025-01-02

**Authors:** Zhaopeng Tong, Chunhua Xing, Xiaomin Xu, Jin‐Jing Xu, Yuanqing Wu, Richard Salvi, Xindao Yin, Fei Zhao, Yu‐Chen Chen, Yuexin Cai

**Affiliations:** ^1^ Department of Otolaryngology Sun Yat‐sen Memorial Hospital Sun Yat‐sen University Guangzhou China; ^2^ Institute of Hearing and Speech‐Language Science Sun Yat‐sen University Guangzhou China; ^3^ Department of Radiology Nanjing First Hospital Nanjing Medical University Nanjing China; ^4^ Department of Otolaryngology Nanjing First Hospital Nanjing Medical University Nanjing China; ^5^ Center for Hearing and Deafness State University of New York at Buffalo Buffalo New York USA; ^6^ Department of Speech and Language Therapy and Hearing Science Cardiff Metropolitan University Cardiff UK

**Keywords:** cognition, network reorganization, neuroimaging, presbycusis

## Abstract

Age‐related hearing loss (ARHL) is considered one of the most common neurodegenerative disorders in the elderly; however, how it contributes to cognitive decline is poorly understood. With resting‐state functional magnetic resonance imaging from 66 individuals with ARHL and 54 healthy controls, group spatial independent component analyses, sliding window analyses, graph‐theory methods, multilayer networks, and correlation analyses were used to identify ARHL‐induced disturbances in static and dynamic functional network connectivity (sFNC/dFNC), alterations in global network switching and their links to cognitive performances. ARHL was associated with decreased sFNC/dFNC within the default mode network (DMN) and increased sFNC/dFNC between the DMN and central executive, salience (SN), and visual networks. The variability in dFNC between the DMN and auditory network (AUN) and between the SN and AUN was decreased in ARHL. The individuals with ARHL had lower network switching rates than controls among global network nodes, especially in the DMN. Some disturbances within DMN were associated with disrupted executive and memory performance. The prolonged loss of sensory information associated with ARHL‐induced compensatory within‐network segregations and between‐network integrations in the DMN might reduce network information processing and accelerate brain aging and cognitive decline.

## Graphical abstract

The authors investigated the spatiotemporal characteristics of brain network functional reorganization in patients with age‐related hearing loss (ARHL) in terms of static and dynamic functional network connectivity and network switching. Compensatory within‐network segregations and between‐network integrations as well as low flexibility in default mode network caused by ARHL are probably linked to cognitive decline, offering new insights on how ARHL contributes to age‐related cognitive impairment.

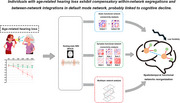

## 1 INTRODUCTION

Age‐related hearing loss (ARHL), a bilateral sensorineural hearing impairment that progresses from high to low‐frequency loss, is the third most common age‐related disability.^1^ This neurodegenerative disorder often leads to difficulties in communication and socialization and can even accelerate the onset of dementia. Recent epidemiological studies suggest that hearing loss is a dominant risk factor for dementia, increasing the risk by 9%.^2^ A cohort study indicated that ARHL precedes the onset of clinical dementia by 5–10 years.^3^ Specifically, adults over the age of 65 with baseline hearing loss develop dementia in an average of 10.3 years, compared to 11.9 years in those with normal hearing.^4^ These findings highlight ARHL as a crucial factor in accelerating the onset of dementia among the elderly. However, the underlying mechanisms linking ARHL and cognitive decline remain poorly understood.

Although hearing aids are widely recommended for ARHL, their efficacy in improving cognitive decline associated with this condition remains uncertain.^5^ Recently, targeted neuromodulation has shown promise in improving cognitive function in Alzheimer's disease and mild cognitive impairment by regulating abnormal functional network interactions.^6^, ^7^ Therefore, investigating the central mechanisms underlying cognitive impairment associated with ARHL from the perspective of functional brain network reorganization is crucial.

Several potential hypotheses have been proposed in the literature to explain their association, including the common cause hypothesis, the information degradation hypothesis, and the sensory deprivation hypothesis.^8^ The common cause hypothesis posits that the concurrent occurrence of cognitive decline and ARHL is due to a shared underlying neurodegenerative pathology.^9^ The information degradation hypothesis suggests that degraded auditory information resulting from ARHL can lead to the reallocation of limited cognitive resources from non‐auditory to auditory cortex.^10^ Lastly, the sensory deprivation hypothesis proposes that ARHL‐induced reorganization of the central auditory pathway, due to long‐term reallocation of cognitive resources, could extend outward, leading to neuroplastic changes in higher level cognitive networks that might result in cognitive deficits.^8^, ^11^, ^12^

Previously, using EEG (Electroencephalogram), we identified abnormal functional changes between specific brain regions in patients with ARHL.^13^ However, cognitive function relies on the spatiotemporal integration of large‐scale, distributed networks that are parallel and interconnected.^14^ Efficient brain network connectivity is essential for optimal brain function. Abnormal interactions between brain networks have been detected in neurological diseases such as schizophrenia,^15^ Alzheimer's disease,^16^ and major depression using neuroimaging methods.^17^ Identifying disturbances within neural networks and disruptions in their functional connections through resting‐state functional magnetic resonance imaging (rs‐fMRI) provides a powerful tool to investigate how ARHL might contribute to cognitive decline.^18^ Several rs‐fMRI studies have demonstrated that organization of the default mode network (DMN), a cognitive‐related network involved in working memory, social cognition, and self‐reference^19^ was generally impaired in ARHL.^20^, ^21^, ^22^ This suggests that long‐term sensory deprivation triggered by ARHL disrupts resting‐state connectivity centered on the DMN. However, most studies have only examined the abnormal changes in functional brain networks associated with ARHL from a static perspective.

Functional connectivity between brain networks is continuously changing even at rest.^23^ These dynamic changes in brain connectivity manifest as specific spatiotemporal patterns that occur alternately and repeatedly, known as large‐scale brain dynamics.^24^ These dynamic interactions within functional networks contribute to cognitive functions such as working memory, executive control, and creativity.^24^, ^25^ The abnormal features of large‐scale brain dynamics reflect the potential mechanisms of neuropsychiatric disorders, including depression,^26^ schizophrenia,^27^ and Alzheimer's disease.^28^ Investigating the dynamic changes in interactions between brain networks can more sensitively identify the aberrant central features associated with cognitive impairment related to ARHL.

Switching between these large‐scale networks is presumed to reflect transitions between brain states involved in different activities.^29^ Recently, multilayer network models have been used to track aberrant network switching in spatiotemporal connectivity patterns of brain nodes comprising non‐overlapping modules in cerebral neurological diseases.^30^ This powerful tool can be utilized to determine if abnormal time‐varying connectivity patterns are associated with cognitive decline linked to ARHL.

The current study aimed to evaluate ARHL‐related widespread functional network features, including (1) static inter‐network functional connectivity, (2) dynamic inter‐network functional connectivity, (3) variability of topological features in functional networks, and (4) network switching rates across whole cerebral regions. Additionally, the study sought to identify disturbances in resting‐state spatiotemporal functional connectivity patterns associated with ARHL and cognitive decline.

## 2 RESULTS

### 2.1 Demographic and clinical characteristics

The demographic and clinical characteristics of individuals with ARHL and healthy controls (HCs) are summarized in Table 1. Individuals with ARHL had a mean age of 59.17 ± 6.52 years and an average educational level of 10.94 ± 1.74 years. There were no significant differences between the groups in terms of age, gender, educational level, the Mini‐Mental State Exam (MMSE), Montreal Cognitive Assessment (MoCA), Auditory Verbal Learning Test (AVLT), Complex Figure Test (CFT), CFT‐delay, clock drawing test (CDT), verbal fluency test (VFT), digit symbol substitution test (DSST), self‐rating depression scale (SDS), and self‐rating anxiety scale (SAS) scores (all *p* > 0.05). Mean pure tone audiometry (PTA) results for both groups are presented in Figure S1, showing significantly higher thresholds in the ARHL group compared to HCs at 0.5, 1, 2, 4, and 8 kHz. Individuals with ARHL also exhibited poorer performance than HCs on Trail‐Making Test A (TMT‐A) (*t* = 2.410, *p* = 0.018), Trail‐Making Test B (TMT‐B) (*t* = 2.357, *p* = 0.020), and DST (*t* = −2.383, *p* = 0.019). These findings indicate that ARHL is associated with worsened executive function and short‐term verbal working memory.

**TABLE 1:** Clinical characteristics of the study subjects.

Characteristics	Individuals with ARHL (*n* = 66)	Healthy controls (*n* = 54)	*t/χ* ^2^	*p* value
Age, years	59.17 (6.85)	57.83 (6.52)	1.084	0.280^a^
Gender, F/M	31/35	32/22	1.799	0.180^b^
Education, years	10.94 (1.74)	10.87 (1.71)	0.218	0.828^a^
PTA of left ear, dB HL	32.82 (4.22)	16.00 (2.81)	26.036	<0.001^c^
PTA of right ear, dB HL	33.05 (6.09)	16.13 (3.23)	19.480	<0.001^c^
Mean PTA of both ears, dB HL	32.93 (3.85)	16.06 (2.27)	29.837	<0.001^c^
MMSE	28.76 (1.14)	28.89 (1.14)	−0.628	0.532^a^
MoCA	26.30 (1.86)	26.83 (1.40)	−1.784	0.077^c^
AVLT	33.02 (6.94)	34.67 (7.17)	−1.278	0.204^a^
CFT	34.65 (1.62)	34.54 (1.65)	0.382	0.703^a^
CFT‐delay	16.45 (3.68)	17.21 (3.70)	−1.132	0.260^a^
TMT‐A	74.48 (23.34)	65.24 (18.68)	2.410	0.018^c^
TMT‐B	172.55 (51.06)	151.11 (47.66)	2.357	0.020^a^
CDT	3.45 (0.53)	3.50 (0.54)	−0.462	0.645^a^
DST	11.00 (1.57)	11.78 (2.01)	−2.383	0.019^a^
VFT	14.30 (3.92)	15.00 (3.69)	−1.006	0.317^a^
DSST	69.06 (7.75)	68.78 (9.48)	0.176	0.860^c^
SAS	36.20 (5.74)	35.44 (6.69)	0.663	0.509^a^
SDS	38.41 (8.05)	36.09 (8.24)	1.552	0.123^a^

*Note*: Values are presented as mean (SD).

Abbreviations: ARHL, age‐related hearing loss; AVLT, Auditory Verbal Learning Test; CDT, clock drawing test; CFT, Complex Figure Test; DSST, digit symbol substitution test; DST, Digit Span Test; F, female; M, male; MMSE, Mini Mental State Exam; MoCA, Montreal Cognitive Assessment; PTA, pure tone audiometry; SAS, self‐rating anxiety scale.; SDS, self‐rating depression scale; TMT‐A, Trail Making Test‐Part A; TMT‐B, Trail Making Test‐Part B; VFT, verbal fluency test.

^a^ Independent two samples *t*‐tests.

^b^ Chi‐squared tests.

^c^ Mann–Whitney *U*‐test.

### 2.2 Aberrant multidimensional functional network in the individuals with ARHL

#### 2.2.1 Functional networks of interest

**FIGURE 1:**
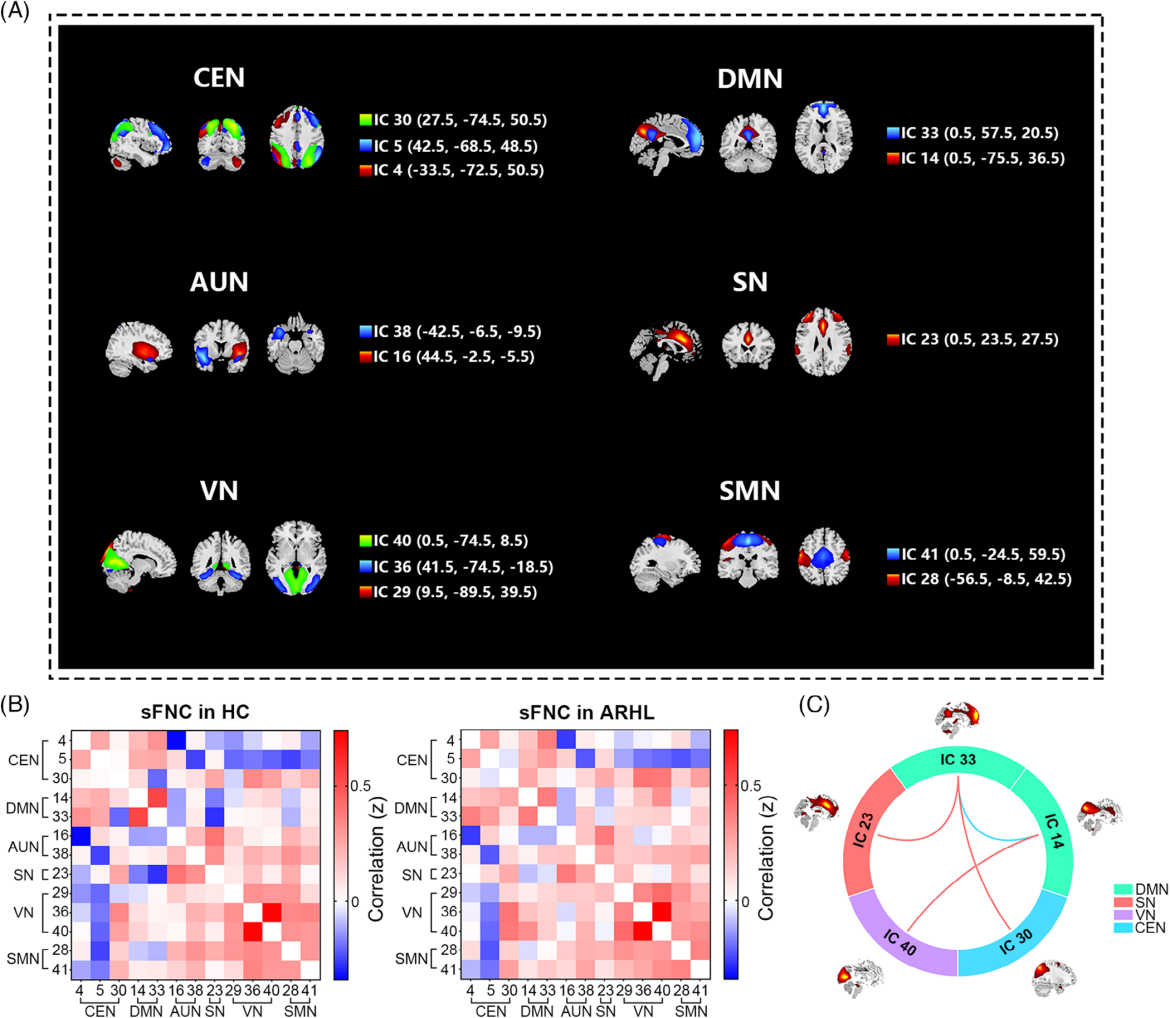
Resting state networks (RSNs) extracted from independent component analysis (ICA) and static functional network connectivity (sFNC) analyses. (A) Spatial maps of 13 independent components (ICs) were selected and divided into several RSNs for further analyses. (B) Subject‐averaged sFNC connectivity matrices from age‐related hearing loss (ARHL) and healthy control (HC). The value in the correlation matrix represents Fisher's *z*‐transformed Pearson correlation coefficient. A positive value is displayed in red, whereas a negative value is displayed in blue. Deeper color means greater absolute value of the correlation coefficient. (C) Significant differences in sFNC network connectivity between the ARHL and the HC groups were controlled for age, gender, and educational level (*q *< 0.05, false discovery rate [FDR] corrected). Stronger connectivity represented by redlines; weaker connectivity shown as blue lines. AUN, auditory network; CEN, central executive network; DMN, default mode network; SMN, sensorimotor network; SN, salience network; VN, visual network.

Figure 1A depicts the selection of 13 independent components (ICs) from a total of 50, classified into six conventional functional networks: the central executive network (CEN), DMN, auditory network (AUN), salience network (SN), visual network (VN), and sensorimotor network (SMN), which were used as networks of interest. The CEN (IC_4_, IC_5_, and IC_30_) included the bilateral dorsolateral prefrontal cortex and posterior parietal cortex. The DMN (IC_4_ and IC_33_) encompassed the bilateral posterior cingulate cortex/precuneus, medial prefrontal cortex, bilateral inferior parietal lobe, and angular gyrus. The AUN (IC_16_ and IC_38_) comprised the bilateral superior and middle temporal gyrus. The SN (IC_23_) encompassed the dorsal anterior cingulate cortex, bilateral insula, and subcortical systems such as the amygdala and ventral striatum. The VN (IC_29_, IC_36_, and IC_40_) mainly included the middle and superior occipital gyrus, temporal–occipital areas, and fusiform gyrus, whereas the SMN (IC_28_ and IC_41_) consisted of the precentral and postcentral gyrus as well as supplementary motor areas.

#### 2.2.2 Static functional network connectivity

The ARHL and HC groups exhibited similar patterns of static functional network connectivity (sFNC). Connections from the CEN and DMN to other functional networks decreased, whereas VN and SMN showed a positive functional intra‐connectivity effect (Figure 1B). Significant differences between the two groups were also observed (Figure 1C). Individuals with ARHL demonstrated increased sFNC in DMN (IC_33_)–SN (IC_23_) (*t* = 3.70, *q_FDR_* = 0.012), DMN (IC_33_)–CEN (IC_30_) (*t* = 3.57, *q_FDR_* = 0.012) and DMN (IC_14_)–VN (IC_40_) (*t* = 4.30, *q_FDR_* = 0.003) but decreased functional network connectivity in DMN (IC_14_)–DMN (IC_33_) (*t* = −3.53, *q_FDR_* = 0.012). Therefore, ARHL is associated with several aberrant static connectivity patterns.

#### 2.2.3 Dynamic functional network connectivity

**FIGURE 2:**
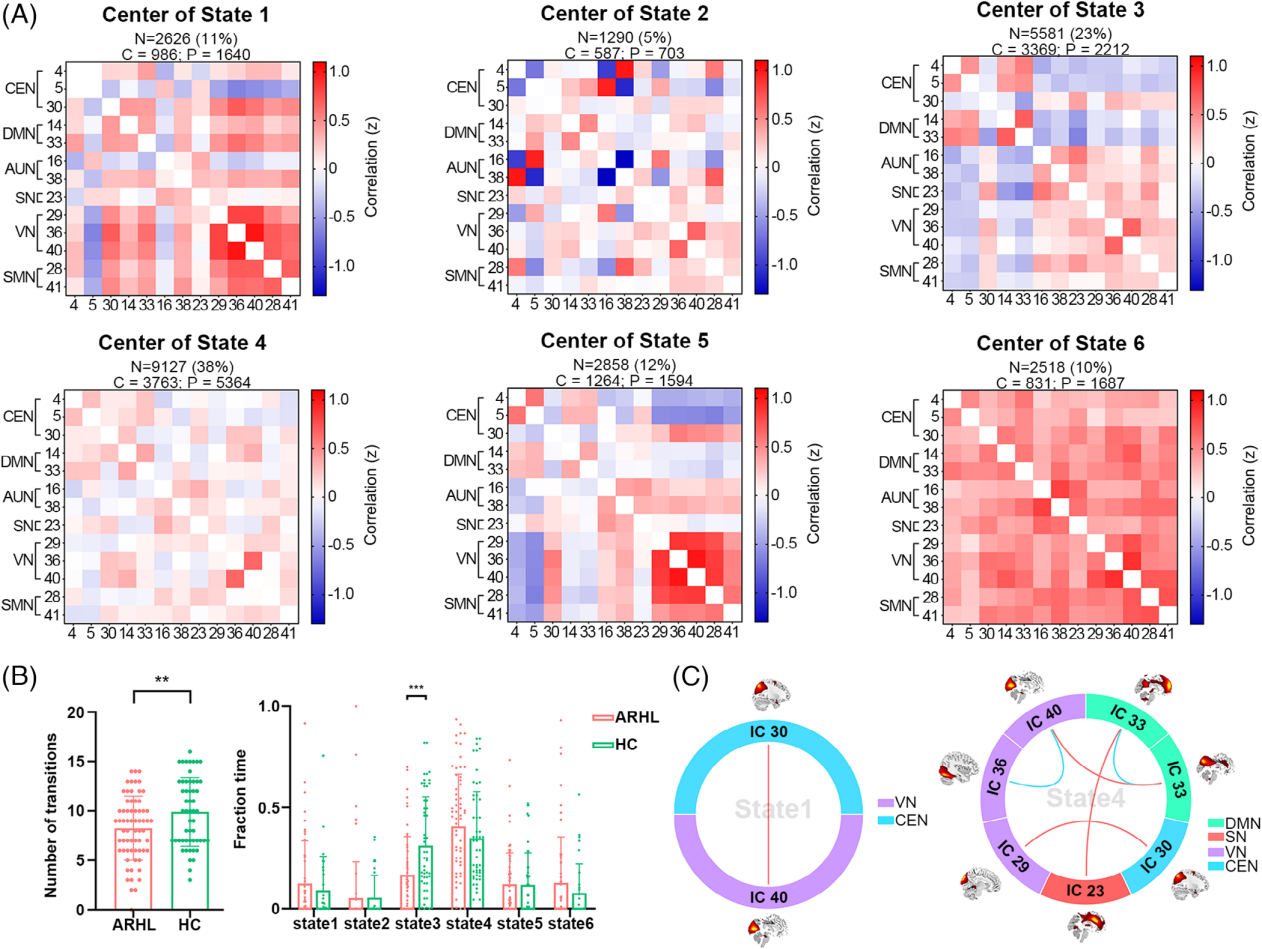
Dynamic functional network connectivity (dFNC) analyses. (A) Cluster centroids, with their occurrence rates (the ratio of the number of the state‐specific windowed dFNC matrices in total ones) and the number of dFNC matrices per group for each state. The value in the correlation matrix represents Fisher's *z*‐transformed Pearson correlation coefficient. A positive value is displayed in red, whereas a negative value is displayed in blue. Deeper color means greater absolute value of the correlation coefficient. (B) Comparisons of number of transitions and fraction time in each state between age‐related hearing loss (ARHL) and healthy control (HC) groups (^**^p < 0.01, ^***^p < 0.001). Each dot denotes a subject's value in the corresponding properties. Error bar denotes the standard deviation. (C) Significant differences of dFNC between ARHL and HC groups in the states, controlled for age, gender, and educational level (*q *< 0.05, false discovery rate [FDR] corrected). Stronger connectivity shown as a redline. Weaker connectivity shown as a blue line. AUN, auditory network; CEN, central executive network; DMN, default mode network; IC, independent component; N, the number of state‐specific windowed dFNC matrices; P, patients with age‐related hearing loss; SMN, sensorimotor network; SN, salience network; VN, visual network.

The sliding‐window dynamic functional network connectivity (dFNC) matrices were clustered into six states using the *k*‐means algorithm (Figure 2A); however, not all subjects exhibited all six states. State 4 had the highest occurrence frequency (38%), characterized by generally weak connectivity among different functional networks. State 3 occurred with a frequency of 23%, where CEN and DMN showed predominantly negative connections to other functional networks. In Figure 2B, it can be observed that the average number of state transitions across all six states was significantly lower in the ARHL group (*t* = −2.68, *p* = 0.008), with State 3 transitions being notably less frequent (*t* = −3.59, *p* = 0.001). Group differences in functional network connectivity within each state are depicted in Figure 2C. In State 1, individuals with ARHL exhibited stronger VN (IC_40_)–CEN (IC_30_) connectivity compared to HC (*t* = 4.34, *q_FDR_* = 0.004). In State 4, individuals with ARHL showed increased DMN (IC_33_)–SN (IC_23_) (*t* = 4.08, *q_FDR_* = 0.003), DMN (IC_14_)–VN (IC_40_) (*t* = 3.11, *q_FDR_* = 0.041), and VN (IC_29_)–CEN (IC_30_) (*t* = 3.49, *q_FDR_* = 0.018) connectivity but decreased VN (IC_36_)–VN (IC_40_) (*t* = −3.07, *q_FDR_* = 0.041) and DMN (IC_14_)–DMN (IC_33_) (*t* = −4.33, *q_FDR_* = 0.003) connectivity.

**FIGURE 3:**
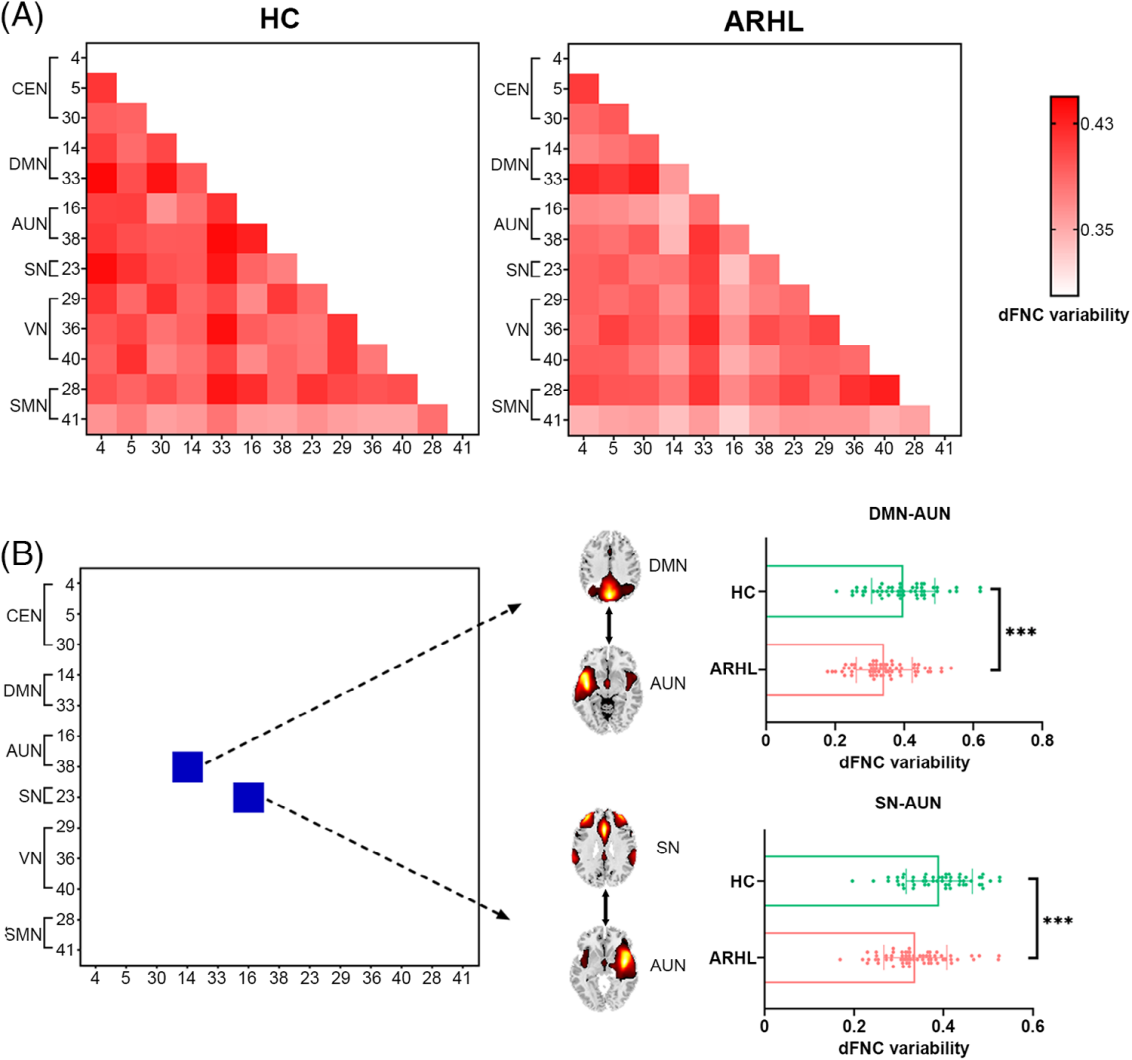
Dynamic functional network connectivity (dFNC) variability analysis. (A) The averaged dFNC variability across the sliding‐window dFNC matrices in the age‐related hearing loss (ARHL) and healthy control (HC) groups. (B) Significant differences of dFNC variability between ARHL and HC groups, controlled for age, gender, and educational level (^*^q < 0.05, ^**^q < 0.01, false discovery rate [FDR] corrected). Each dot denotes a subject's dFNC variability value. Error bar denotes the standard deviation. AUN, auditory network; CEN, central executive network; DMN, default mode network; IC, independent component; SMN, sensorimotor network; SN, salience network; VN, visual network.

The strength of functional connectivity varied over time, as indicated by the mean variability of dFNC across the six matrices (Figure 3A). Individuals with ARHL showed significantly decreased variability in DMN–AUN (*t* = −3.39, *q_FDR_* = 0.036) and SN–AUN (*t* = −4.43, *q_FDR_* = 0.002) compared to HC (Figure 3B). Dynamic graph theory analysis, based on the variance of dFNC matrices from each subject, revealed no significant differences in global efficiency and local efficiency flexibility between the two groups.

#### 2.2.4 Network switching rate

**FIGURE 4:**
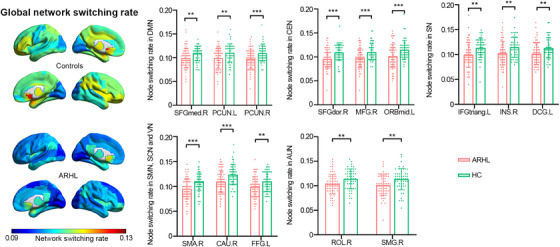
Multilayer network analysis. The left side shows the configurations of global network switching rates and network switching rates on the cerebral surface in the individuals with age‐related hearing loss (ARHL) and healthy controls (HCs). The bar charts exhibited significant differences of network switching rates in different functional networks in the ARHL and HC groups controlled for age, gender, and educational level (^*^q < 0.05, ^**^q < 0.01, false discovery rate [FDR] corrected). AUN, auditory network; CAU.R, right caudate nucleus; CEN, central executive network; DCG.L, median cingulate and paracingulate gyrus; DMN, default mode network; FFG.L, left fusiform gyrus; IFGtriang.L, left triangular inferior frontal gyrus; INS.R, right insula; MFG.R, right middle frontal gyrus; ORBmid.L, left orbital middle frontal gyrus; PCUN.L, left precuneus; PCUN.R, right precuneus; ROL.R, right Rolandic operculum; SCN, subcortical network; SFGdor.R, right dorsolateral superior frontal gyrus; SFGmed.R, right medial superior frontal gyrus; SMA.R, right supplementary motor area; SMG.R, right supramarginal gyrus; SMN, sensorimotor network; SN, salience network; VN, visual network.

Figure 4 depicts the global network switching rates across the cerebral cortex. Visual inspection of the data reveals a marked decrease in global network switching rates in the ARHL group compared to the HC group. Specifically, nodes within these extensive functional networks exhibited significantly lower network switching rates in individuals with ARHL compared to HC (as shown in Figure 4 and Table S1).

### 2.3 Head motion effect control and validated results

As described in the Method section, head motion analysis revealed no significant difference in mean framewise displacement (FD) between the two groups (ARHL: 0.24 ± 0.06 mm; HC: 0.22 ± 0.08 mm; *p* = 0.178). Controlling for mean FD as a covariate, most significant results remained valid (sFNC: 4/4; dFNC: 4/6; network switching rate: 12/14, as shown in Tables S2–S4). These findings indicate that head motion minimally influenced the outcome measures in this study.

### 2.4 Correlation between clinical traits and network metrics

To facilitate the interpretation of the fMRI results, correlation analyses were conducted to investigate whether any of the multidimensional network metrics were associated with the clinical traits that distinguished individuals with ARHL from HC. Regarding sFNC, sFNC in DMN (IC_14_)–DMN (IC_33_) showed a negative correlation with TMT‐B, as illustrated in Figure S2A. In terms of dFNC, dFNC in DMN (IC_14_)–DMN (IC_33_) during State 4 exhibited a negative correlation with TMT‐A (Figure S2B). Additionally, for modular variability, network switching rates in the left precuneus were positively correlated with DST, and network switching rates in the right precuneus were also positively correlated with DST (Figure S2C). There was no significant correlation between the mean hearing thresholds at 0.5, 1, 2, and 4 kHz and the abnormal functional network characteristics in individuals with ARHL after false discovery rate (FDR) correction (all *q_FDR_ *> 0.05, Tables S5–S8).

### 2.5 Correlation among different network metrics

In individuals with ARHL, the relationships among different network metrics were also assessed. Within sFNC, significant positive correlations were observed between CEN–DMN, DMN–VN, and SN–DMN sFNC (refer to Table S9). Regarding state properties, the fraction of time spent in State 3 showed negative correlations with CEN–DMN, DMN–VN, and SN–DMN sFNC but exhibited a positive correlation with DMN–DMN sFNC (refer to Table S10). More detailed results of the correlations between network switching rates and sFNC and dFNC are provided in Tables S11–S15.

### 2.6 Subgroup differences on abnormal functional network features in the individuals with ARHL

According to the MoCA scoring classification criteria, the ARHL group with cognitive decline consisted of 30 individuals, whereas the ARHL group without cognitive decline consisted of 36 individuals. There were no significant differences in abnormal functional network characteristics between these two groups (all *q_FDR_* > 0.05, see Tables S16–S20). Similarly, based on age classification criteria, there were 38 individuals in the older group and 28 in the younger group. No significant differences were observed in abnormal functional network reorganization caused by ARHL between these age groups (all *q_FDR_* > 0.05, see Tables S16–S20).

## 3 DISCUSSION

Typical interactions between brain functional networks support normal cognitive function,^14^ whereas aberrant reorganization of these networks may contribute to cognitive impairment associated with ARHL.^31^ To test this hypothesis, we investigated altered interactions of brain functional networks in patients with ARHL. We identified abnormal static and dynamic inter‐ and intra‐connections between the DMN and other functional networks, as well as decreased network switching rates among key nodes within the DMN in individuals with ARHL. Consistent with our hypothesis, several of these changes in functional networks correlated with specific cognitive deficits.

The DMN is situated at the culmination of processing streams originating from cortices involved in various high‐order cognitive activities.^32^ Contrary to traditional beliefs of attenuated activity, the DMN is increasingly recognized for its activation when behaviors draw upon prior experiential information,^33^ integrating incoming extrinsic information with existing intrinsic knowledge.^34^ Prior experience plays a critical role in accurate speech comprehension,^35^ particularly in conditions where auditory input is degraded.^36^ The heightened connectivity observed between the DMN and other functional networks in individuals with ARHL likely stems from their reliance on prior knowledge to comprehend degraded speech resulting from peripheral hearing loss. Furthermore, our study demonstrates increased between‐network integrations involving the DMN in individuals with ARHL, suggesting that compensatory mechanisms involving the CEN, SN, and VN are engaged to support normal cognitive function in the face of degraded auditory and semantic information.^14^, ^34^, ^37^

Significant network disturbances were observed in individuals with ARHL who exhibited cognitive decline.^10^ One potential downside of the heightened functional network connectivity seen in ARHL is that it disrupts network segregation, thereby limiting the information processing capacity that networks such as the CEN, SN, and VN can allocate to other functions like execution and attention.^38^, ^39^ The positive correlations found between increased functional connectivity of DMN–CEN, DMN–VN, and DMN–SN suggest that compensatory recruitment of DMN resources may underlie the increased network integrations observed in individuals with ARHL. However, an intriguing possibility is that the abnormal recruitment and integration of the DMN into these networks could potentially mask the cognitive impairments associated with ARHL.

The DMN is recognized as a highly interconnected brain network^40^; however, the functional connectivity between its anterior and posterior regions decreases in advanced normal aging.^41^, ^42^ This reduced within‐network connectivity in the DMN could potentially indicate that the brain is experiencing accelerated aging due to ARHL. The weakening of long‐distance connections between the anterior and posterior DMN regions appears to impair functional brain maturation and diminish network efficiency, thereby compromising cognitive reserve. Furthermore, the diminished connection between the anterior and posterior DMN is associated with declines in executive function,^43^ suggesting a possible mechanism for cognitive impairment in ARHL. The normal interaction between these DMN regions maintains the integrity of internal experiences, such as self‐referential processing, episodic or autobiographical memory, and future thinking, integrating relevant external and internal information to guide thoughts and behaviors.^44^ Therefore, disruptions in information flow between these midline structures may interfere with this guiding mechanism, leading to impaired executive abilities.

Neurodegenerative conditions frequently disrupt the dynamic flexibility of the brain system.^31^, ^45^ In this study, ARHL induced aberrant dynamic features of functional network connectivity. According to the dFNC results, individuals with ARHL exhibited significantly fewer transitions between states compared to HCs. Cabral et al. demonstrated that healthy older adults with decreased state transitions exhibited poorer cognitive performance.^46^ The reduced number of state transitions in ARHL is associated with decreased brain perfusion, thereby reducing the efficiency of information processing in the cerebral cortex.^45^ Moreover, matrices corresponding to States 3 and 4 collectively represented more than half of the total transitions. Thus, these two brain states likely play pivotal roles in cerebral function. State 4, being the most prevalent brain state, exhibited notably weak inter‐ and intra‐network connectivity in ARHL. This specific connectivity pattern is also prominently observed in the elderly^45^ and has been reported to increase in frequency in Alzheimer's disease and other neurodegenerative disorders,^31^, ^47^ where prolonged time spent in weakly connected states correlates with poorer cognitive function.^45^

Compared to HCs, individuals with ARHL tended to spend more time in State 4. In this state characterized by weak network associations, ARHL individuals exhibited a dysconnectivity pattern within and between networks similar to sFNC. This aligns with findings of negative correlations between decreased functional network connectivity within DMN and executive performance, suggesting accelerated aging and compensatory alterations due to degraded auditory input leading to cognitive impairment. State 3, on the other hand, demonstrated strong within‐network connectivity but weak between‐network connections in CEN and DMN, resembling a normal physiological state.^20^, ^45^ However, ARHL participants showed a shorter fraction time (FT) in State 3. This shorter time correlated negatively with abnormal CEN–DMN, VN–DMN, and SN–DMN sFNC, implying that individuals with less time in State 3 may recruit more cognitive resources to DMN, potentially weakening information processing efficiency in other networks. Furthermore, the positive correlation between FT in State 3 and DMN–DMN sFNC suggests that reduced State 3 occupation may disrupt information transmission between anterior and posterior DMN, contributing to cognitive frailty.^48^ Therefore, although the connection pattern of State 3 appears beneficial for cognitive function, it seems compromised in ARHL due to detrimental compensatory mechanisms.

Connection variability is considered indicative of the complexity of information processing efficiency.^26^ Regions such as the precuneus and angular gyrus within the posterior DMN play critical roles in integrating auditory and visual inputs essential for high‐order cognition.^32^, ^49^ The SN is activated to guide behaviors using internal cues and external information, mediated by the anterior cingulate cortex, which integrates inputs from auditory and visual cortices.^50^, ^51^ ARHL is likely to reduce the relay of auditory information from the auditory cortex to the DMN and SN, contributing to decreased connection variability. Moreover, the diminished connection variability in ARHL suggests reduced efficiency in information processing within the DMN and SN, possibly due to prolonged compensatory mechanisms aimed at deciphering degraded speech signals.^37^, ^52^

In the current study, dynamic graph theory analysis revealed no significant differences in global efficiency and local efficiency flexibility between the two groups. Global efficiency and local efficiency flexibility are indicative of fluctuations in brain network integration and segregation. Our findings from FNC analyses highlighted within‐network segregations and between‐network integrations in the DMN among individuals with ARHL. These results suggest that despite the network reconfiguration observed in ARHL, the overall network stability remained relatively stable.

Whole‐brain network switching rates reflect the dynamic flexibility of multiple functional networks, crucial for high‐order cognitive functions like working memory, planning, and reasoning.^30^ Whole‐brain network switching rates reflect the dynamic flexibility of multiple functional networks, crucial for high‐order cognitive functions like working memory, planning, and reasoning.^53^ The decreased network‐switching rate observed in supplementary motor areas of individuals with ARHL may be associated with impaired physical performance linked to dementia,^54^ possibly due to long‐term resource recruitment effects induced by hearing loss impeding interactions between the SMN and other brain networks. The precuneus extensively integrates and processes visual and auditory information.^55^ Furthermore, it maintains robust structural connections with the hippocampus, underscoring its role in diverse memory functions, including visual and verbal working memory.^55^, ^56^ The lower network switching rate observed in the bilateral precuneus diminishes the efficiency of information processing within the DMN in individuals with ARHL. Therefore, these findings suggest that ARHL could contribute to impaired auditory information processing in the precuneus, thereby potentially compromising verbal working memory capacity in individuals affected by ARHL.

However, contrary to previous studies on ARHL,^22^, ^57^ the extent of abnormalities in functional network organization due to ARHL did not show a significant association with the severity of hearing loss in the individuals included in our study. One possible explanation for this finding could be that all participants with ARHL in our study had mild hearing loss. The observed changes in abnormal functional network interactions may thus reflect the specific characteristics of functional reorganization occurring in response to mild hearing loss.

The subgroup analysis results indicated that neither age nor cognitive ability, as measured by MoCA, had a significant effect on the neural network reorganizations observed in individuals with ARHL in our current study. This finding contrasts somewhat with previous research.^21^ One plausible explanation could be that the limited sample size after subgrouping may have reduced the statistical power to detect significant effects. However, it also suggests that our findings are not confounded by variations in cognitive ability or age, highlighting ARHL as an independent factor influencing central neural network reorganization. Moreover, these results imply that ARHL may lead to brain network changes in relatively younger individuals with better cognitive function that resemble those seen in older individuals with poorer cognitive function. This suggests a potential role for ARHL in accelerating brain aging and impairing cognitive function. In future studies, expanding the sample size and further subgrouping individuals with ARHL based on different factors will allow for a deeper exploration of how these variables influence neural network reorganization.

The study was limited by the lack of participants with varying severities of hearing loss. Consequently, the results only represent the large‐scale functional network reorganization in individuals with ARHL who have mild hearing loss, as indicated by their mean thresholds at 0.5, 1, 2, and 4 kHz. To enhance the efficacy of our experimental design, future research should include a larger number of participants and incorporate comparisons among individuals with ARHL who exhibit substantially different degrees of hearing loss. Additionally, we did not investigate the duration of ARHL among participants in this study. Considering the potential impact of hearing loss duration on whole‐brain functional networks and cognitive function, it is insightful to further the underlying relationships between the duration of ARHL and neural network reorganization. Regarding sample size, we did not evaluate it based on the effect sizes from previous studies. To improve the reliability of study results, future fMRI studies on ARHL will calculate sample sizes based on the effect size from the current study. Furthermore, due to the limited sample size, the results of cognitive and age‐related subgroup analyses should be interpreted cautiously. A within‐subject longitudinal design would be a more powerful approach to elucidate if and how ARHL contributes to cognitive decline and network dysfunction. However, the slow progression of ARHL makes it difficult and costly to accumulate imaging data from a large sample of individuals with ARHL. Finally, ARHL was quantified using only pure‐tone audiometry, which captures limited information about hearing disorders. Further assessments related to central auditory processing, such as speech intelligibility tests, could provide a better understanding of the potential role ARHL plays in cognitive impairment.^8^

Our results demonstrate that ARHL disrupts specific aspects of resting‐state functional connectivity patterns across the frontal–parietal regions of the central nervous system. These changes likely reflect cortical reorganization resulting from auditory sensory deprivation. The compensatory within‐network segregations and between‐network integrations in the DMN presumably disrupt network information processing, thereby accelerating brain aging and contributing to cognitive decline. The altered connectivity patterns associated with ARHL and their implications for cognitive decline may provide new insights into how ARHL contributes to age‐related cognitive impairment. Moreover, our findings suggest that regions associated with the DMN can serve as neural modulation targets to improve cognitive impairment related to ARHL.

## 4 MATERIALS AND METHODS

### 4.1 Participants

Sixty‐six individuals with ARHL and 54 HCs closely matched for age‐, gender‐, educational level‐, and handedness were recruited from the community health center or online advertisements between January 2019 and December 2021. The details for the determination of the sample size were shown in Method S1.

Individuals with ARHL met the following inclusion criteria: (1) average hearing threshold (0.5, 1, 2, and 4 kHz) above 25 dB HL in the better hearing ear; (2) 50–80 years old; (3) type A tympanogram; (4) right‐handed. Eligible HCs had hearing within normal limits with average hearing threshold (0.5, 1, 2, and 4 kHz) below 25 dB HL. The inclusion criteria for controls (age, type of tympanogram, and handedness) were matched to the individuals with ARHL. Exclusion criteria for all subjects were as follows: (1) other ear diseases potentially affecting hearing threshold, such as tinnitus, Meniere's disease, and otosclerosis, (2) a past history of occupational noise exposure, hearing aid use, otologic surgery, ototoxic drug use, cerebral trauma, and epilepsy, (3) mental or neurological disorders, (4) major illnesses (e.g., anemia, cancer, and intracranial infection), (5) smoking or alcohol addiction, and (6) contraindications to magnetic resonance imaging (MRI).

### 4.2 Auditory assessment

A series of clinical assessments were included to evaluate subject's audiological and cognitive situation, such as PTA, tympanometry, cognitive, and psychological assessments. PTA was performed in an acoustically shielded room and a professional audiologist assessed subject's air conduction hearing thresholds at 0.25, 0.5, 1, 2, 4, and 8 kHz on bilateral ears (more details see Method S2). In addition, tympanometry was measured by a Madsen Electronics Zodiac 901 Middle Ear Analyzer (GN Otowrics) for the evaluation of the middle ear function.

### 4.3 Neuropsychological assessment

To evaluate various cognitive domains, such as memory, attention, executive function, and visuospatial ability, a set of cognitive assessments was conducted. These assessments included: MMSE and MoCA for general cognitive ability; AVLT for verbal memory processes; CFT for immediate and delayed visual memory recall; DST for short‐term working memory; TMT‐A and TMT‐B for executive function; CDT for executive function and visuospatial function; DSST for motor speed, attention, and visuoperceptual functions and VFT for semantic memory. Additionally, SDS and SAS were used to evaluate the degree of depression and anxiety for each subject. Detailed information on these measures is provided in Method S3.

**FIGURE 5:**
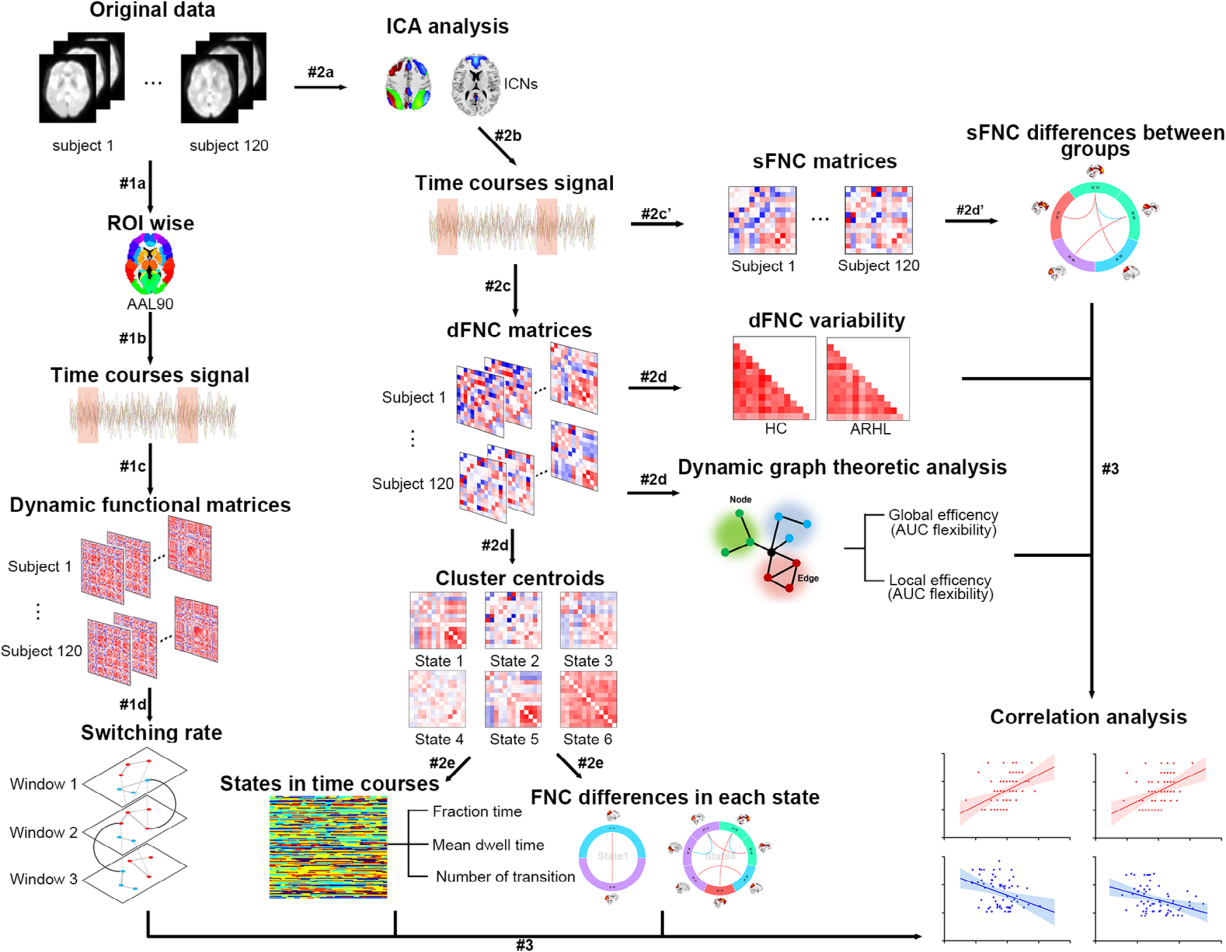
Flowchart of resting‐state functional magnetic resonance imaging (rs‐fMRI) analyses. The original data were processed by three analyses that included a multilayer network analysis (#1), a static and dynamic functional network connectivity (dFNC) analysis (#2), and a correlation analysis (#3). For the multilayer network analysis, a series of sliding‐window functional matrices were extracted from preprocessed fMRI signals using the AAL 90 atlas (#1a, #1b, and #1c). Then an iterative and ordinal Louvain algorithm was used to track time‐dependent alterations in these functional networks and the network‐switching rate of each node in the AAL 90 atlas (#1d). For the static FNC (sFNC) and dFNC analyses, an independent component analysis (ICA) was performed on the preprocessed fMRI signal (#2a) in order to reduce the data dimension into a set of independent components (ICs) (#2b). The ICs were categorized into six classic functional networks, default mode network (DMN), central executive network (CEN), salience network (SN), visual network (VN), sensorimotor network (SMN), and auditory network (AUN). Pearson correlations were calculated between summary time course network‐pairs for sFNC analysis (#2c′). Further group‐level differences on the sFNC were evaluated (#2d′). Pearson correlations between network‐pairs were calculated for windowed dFNC matrices in each subject based on the sliding window approach (#2c). Afterward, the dFNC matrices were clustered into several states by the *k*‐means algorithm and dFNC variability and dynamic graph theory flexibility were calculated across the sliding windows (#2d). The parameters and group‐level differences on functional connectivity of each state were estimated (#2e). All group‐level rs‐fMRI indicators from healthy control (HC) and age‐related hearing loss (ARHL) were compared. The correlations were calculated between various clinical traits and rs‐fMRI indicators as well as between different rs‐fMRI indicators (#3). AAL, automated anatomical labeling; AUC, area under curve; ROI, region of interest.

The normality of all demographic variables was evaluated using the Shapiro–Wilk test. For group‐level comparisons, continuous variables were assessed with two‐sample *t*‐tests, whereas categorical variables were evaluated using chi‐square tests. Variables were considered statistically significant when *p* < 0.05. All statistical analyses were performed using SPSS, version 25.0 (SPSS). An overview of the experimental design is shown in Figure 5.

### 4.4 Imaging data acquisition and preprocessing

All MRI data were obtained using a 3.0 T Philips MRI scanner (Ingenia) equipped with an eight‐channel phased‐array head coil. During scanning, subjects were instructed to lie quietly and remain as still as possible with their eyes closed, avoiding sleep or any specific thinking activities. Foam padding and earplugs were utilized to minimize head movement and reduce the disturbance caused by scanning noise. The earplugs (Hearos Ultimate Softness Series) attenuate scanner noise by approximately 32 dB. fMRI data were collected using a gradient echo‐planar imaging sequence that lasted for 8 min and 8 s, with the following parameters: slice thickness = 4 mm, number of slices = 36, gap = 0 mm, repetition time (TR) = 2000 ms, echo time = 30 ms, flip angle = 90°, matrix size = 64 × 64, and field of view = 240 mm × 240 mm. The voxel size was 3.75 × 3.75 × 4.00 mm^3^. Scans were obtained using parallel imaging with the sensitivity encoding (SENSE) technique, and the SENSE factor was 2.

Functional data preprocessing was conducted using the Graph Theoretical Network Analysis Toolbox for Imaging Connectomics (GRETNA, version 2.0.0; http://www.nitrc.org/projects/gretna/).^58^ The specific preprocessing steps included: (1) removal of the first 10 volumes; (2) slice‐timing correction (using the middle slice as the reference slice); (3) realignment (registering to the first image; any individuals with head motion > 2.0 mm translation or rotation angle > 2.0° in any direction, or a mean FD > 0.5 mm, were excluded from subsequent analysis); and (4) spatial normalization to the Montreal Neurological Institute template with resampling voxel size = 3 × 3 × 3 mm^3^ and smoothing with a 6 × 6 × 6 mm^3^ full‐width at half‐maximum Gaussian kernel.

### 4.5 Group independent component analysis

Group‐level spatial independent component analysis (ICA)^59^ was performed using GIFT software (version 4.0b, http://mialab.mrn.org/software/gift/) to decompose the preprocessed fMRI data into distinct functional networks. The ICA procedure involved several steps: data dimensionality reduction, application of the ICA algorithm, and backward reconstruction for each subject (see Method S4 for details). Using an Infomax algorithm following a two‐step principal component analysis, 50 ICs were extracted. The number of the ICs was estimated using the minimum description length criteria. Based on criteria for selecting resting‐state networks, 13 ICs were chosen through visual inspection and were categorized into 6 conventional functional networks: DMN, CEN, SN, AUN, VN, and SMN. The selection of these networks was based on spatial maps and atlases used in previous studies. More detailed selection procedures are described in Method S4.

### 4.6 Static and dynamic FNC analysis

The MANCOVAN module in GIFT software was used for sFNC analysis. Pearson correlation coefficients between pairwise ICs were calculated using the summary time course of each selected IC, representing the strength of FNC. For further analysis, the correlation coefficients were transformed using Fisher's *z*‐scores. This transformation generated a 13 × 13 IC correlation matrix for each subject.

To reveal the dynamic characteristics of FNC, the temporal dFNC toolbox in GIFT software was employed. A sliding‐window method was used to extract FNC signals over time from the total time course, allowing for the evaluation of changes in FNC between pairs of ICs. This method utilized a rectangular window size set to 20 TRs, convolved with a Gaussian (*σ* = 3), and advanced in steps of 1 TR. Details related to the choice of the window size are provided in Method S5. A series of 3D (13 components × 13 components × 200 windows) windowed FNC matrices were generated for each subject, representing the dynamic features of FNC between functional networks. The windowed FNC matrices were automatically clustered into six separate states using the *k*‐means algorithm (Method S5). From these matrices, three temporal properties of dFNC states were extracted for each subject: FT, mean dwell time of each state, and the number of transitions between states. Additionally, dFNC variability, an indicator of FNC network stability, was defined as the standard deviation of the strength of FNC (Pearson's correlation coefficients) between pairs of ICs across windows.^26^

### 4.7 Dynamic network topology analysis

To assess the variability of topological metrics within the functional connectivity network, we defined 13 ICs as nodes and used Pearson correlations between these components as edges. Undirected sparse binary matrices were created following a standard pipeline, as described in Method S6. In this study, two topological properties were calculated at each sparsity threshold: global efficiency and local efficiency. These measures reflect the efficiency of parallel information transfer between global nodes and neighboring nodes within the functional networks, respectively.^60^ Differences in the variability of global and local efficiency between individuals with ARHL and HCs were then determined (see Method S6).

### 4.8 Multilayer modularity and network switching analysis

In the current study, the multilayer network, consisting of 201 time‐ordered functional network windows for each subject (see Method S7), incorporates functional connectivity information within a layer (i.e., window) and between adjacent layers, reflecting the dynamic changes in brain network modularity. Compared with randomly distributed connections, a set of nodes within a community has stronger connections to each other than to those outside the community. Modularity is a process designed to find an optimal number of communities in a brain network using an iterative algorithm.^61^

An iterative and ordinal Louvain algorithm was used for optimal modularity detection across the multilayer network.^30^ Two parameters were set to *γ* = 1.1 and *ω* = 0.5; the detailed modularity measurement is shown in Method S7.^30^ The dynamic modular information of the 90 nodes in each window (i.e., dynamic functional matrix) over time was estimated for each subject. Consequently, the percentage of time windows in which a node changes from one community to another was calculated. This metric was defined as the network‐switching rate for a node.

Above different fMRI variables were compared using a general linear model (GLM) with age, gender, and educational level as covariates. Except for the temporal properties of dFNC states, all other significance levels were corrected by FDR (*q* < 0.05) using the Benjamini–Hochberg method.^62^ The FDR corrections were calculated using the fdr_bh function in MATLAB (https://www.mathworks.com/matlabcentral/fileexchange/27418‐fdr_bh).

### 4.9 Correlation analysis

Correlations between the multidimensional network metrics and clinical traits were investigated using partial correlation analysis. This analysis was adjusted for age, gender, and educational level to control for potential confounding factors. The relationships between pairs of network metrics were also evaluated to understand the interplay among different network characteristics. Additionally, the effect of the severity of hearing loss on aberrant brain network features in individuals with ARHL was explored. Partial correlation analysis was used to calculate the relationships between the severity of hearing loss and aberrant brain network features, also adjusting for age, gender, and educational level. Statistical significance for all correlation analyses was set to FDR‐corrected *q* < 0.05.

### 4.10 Head motion control and validation analysis

To minimize the effect of head motion on functional network analyses, individuals with head motion greater than 2.0 mm translation, rotation angle greater than 2.0°, or mean FD greater than 0.5 mm were excluded from the study. Additionally, to further account for the potential effects of head motion, the mean FD was compared between the two groups using a two‐sample *t*‐test. The significant results of sFNC, dFNC, and network switching rate were then validated by controlling for mean FD as one of the covariates in the analysis.

### 4.11 Cognition and age‐related subgroup analysis

To investigate the impact of cognitive function and age on neural networks, subgroup analyses of the individuals with ARHL were conducted based on MoCA scores and age. The individuals with ARHL were categorized using MoCA ≥ 26 or age ≥ 60 as the cut‐off points, respectively. The differences in the aberrant functional network features found in individuals with ARHL were compared between the ARHL with cognitive decline (MoCA < 26) group and the ARHL without cognitive decline (MoCA ≥ 26) group, as well as between the older group (age ≥ 60) and the younger group (age < 60). These comparisons were performed using a GLM, controlling for age, gender, and educational level as covariates. All comparisons were corrected for the FDR using the Benjamini–Hochberg FDR method.

## AUTHOR CONTRIBUTIONS

**Zhaopeng Tong**: Conceptualization; methodology; formal analysis; investigation; writing—original draft; visualization. **Chunhua Xing**: Conceptualization; methodology; formal analysis; investigation; writing—original draft; visualization. **Xiaomin Xu**: Data curation; methodology; investigation. **Jin‐Jing Xu**: Data curation; investigation. **Yuanqing Wu**: Data curation; investigation. **Richard Salvi**: Investigation; methodology; writing—review and editing. **Xindao Yin**: Investigation; methodology; writing—review and editing. **Fei Zhao**: Investigation; methodology; writing—review and editing. **Yu‐Chen Chen**: Funding acquisition; project administration; resources; writing—review and editing; conceptualization. **Yuexin Cai**: Funding acquisition; project administration; resources; writing—review and editing; conceptualization. All authors have read and approved the final manuscript.

## CONFLICT OF INTEREST STATEMENT

The authors declared no potential conflicts of interest.

## ETHICS STATEMENT

The study protocol was approved by the Medical Research Ethics Committee of Nanjing Medical University (KY2021000589YX), and written informed consent was obtained from each subject.

## Supporting information



Supporting Information

## Data Availability

The datasets used or analyzed during the current study are available from the corresponding author on reasonable request.
