# Supplementary material for: Impaired network organization in mild age‐related hearing loss
Source: MedComm (2020). 2025 Jan 2;6(1):e70002. doi: 10.1002/mco2.70002 (PMC11695200; doi:10.1002/mco2.70002)
Supplement: Supplementary file 1 — Supporting Information [file MCO2-6-e70002-s001.docx]

**Supplementary materials**

**Title:** Impaired network organization in mild age-related hearing loss

**Zhaopeng Tong et al**

The supplementary materials are listed as follows:

**Method S1.** Group independent component analysis

**Method S2.** Static and Dynamic FNC analysis

**Method S3.** Dynamic network topology analysis

**Method S4.** Multilayer modularity and network switching analysis

**Fig. S1.** Mean hearing threshold in individuals with ARHL and healthy controls

**Fig. S2.** Correlations between clinical traits and network metrics

**Table S1.** Between-group comparisons for node switching rate in different functional networks

**Table S2.** Validation analysis for significant differences on static FNC between ARHL patients and healthy controls after controlling for the mean FD

**Table S3.** Validation analysis for significant differences on dynamic FNC between ARHL patients and healthy controls after controlling for the mean FD

**Table S4.** Validation analysis for significant differences on network switching rate between ARHL patients and healthy controls after controlling for the mean FD

**Table S5.** Correlations between the severity of hearing loss and abnormal static functional network connectivity in individuals with ARHL

**Table S6.** Correlations between the severity of hearing loss and abnormal dynamic metrics in individuals with ARHL

**Table S7.** Correlations between the severity of hearing loss and abnormal dynamic functional network connectivity in state 4 in individuals with ARHL

**Table S8.** Correlations between the severity of hearing loss and abnormal dynamic functional network connectivity variability in individuals with ARHL

**Table S9.** Correlation within aberrant sFNC in patients with ARHL

**Table S10.** Correlation between abnormal sFNC and fraction time in state 3 in patients with ARHL

**Table S11.** Correlation between aberrant network switching rate and fraction time in state 3 in patients with ARHL

**Table S12.** Correlation between aberrant sFNC and abnormal network switching rate in DMN in patients with ARHL

**Table S13.** Correlation between aberrant dFNC variability and abnormal network switching rate in patients with ARHL

**Table S14.** Correlation between aberrant dFNC in state 4 and abnormal network switching rate in patients with ARHL

**Table S15.** Correlation between aberrant dFNC in state 4 and abnormal network switching rate in patients with ARHL

**Table S16.** Cognition- and age-related subgroup analyses: between-group comparisons for aberrant static functional network connectivity in the individuals with ARHL

**Table S17.** Cognition- and age-related subgroup analyses: between-group comparisons for aberrant dynamic functional connectivity in state 4 in the individuals with ARHL

**Table S18.** Cognition- and age-related subgroup analyses: between-group comparisons for aberrant dynamic network metrics in the individuals with ARHL

**Table S19.** Cognition- and age-related subgroup analyses: between-group comparisons for aberrant dynamic functional connectivity variability in the individuals with ARHL

**Table S20.** Cognition- and age-related subgroup analyses: between-group comparisons for aberrant network switching rate in the individuals with ARHL

**Table S21.** The sensitivity of the cognitive performance measures for detecting cognitive related changes used in the current study

**Supplementary methods**

**Method S1. Participants**

We did not evaluate the sample size based on the effect size from previous studies. The sample size for this study was determined based on the following three principles: First, the sample size should reach the typical level of previous fMRI studies (the median sample size per group in previous 10 years fMRI studies is between 11 and 12.5)^1^. Second, building on the first point, each group should have a sample size of at least 40^2^. Third, considering the cost, the larger the sample size, the higher the statistical power, and the more reliable the results.

**Method S2. Auditory assessment**

The PTA was conducted on a clinical audiometer Interacoustic AD229e using TDH-39 headphones (Interacoustics, Denmark, Audiometer Alle, 5500 Middelfart) previously calibrated according to ISO 389-1: 1998. This measurement was conducted by means of conventional 10 dB down and 5 dB up bracketing method (modified Hughson-Westlake method) in accordance with the recommendations of the British Society of Audiology (https://www.thebsa.org.uk/wp-content/uploads/2023/10/OD104-32-Recommended-Procedure-Pure-Tone-Audiometry-August-2018-FINAL-1.pdf.).

**Method S3. Neuropsychological assessment**

The Mini-Mental State Exam (MMSE) is a widely used 30-point questionnaire designed to assess cognitive impairment. It encompasses a variety of tasks across several domains: orientation to time and place, repetition of word lists, arithmetic tasks such as serial sevens, language use and comprehension, and basic motor skills^3^. The Montreal Cognitive Assessment (MoCA) is a 30-point questionnaire that evaluates eight cognitive domains. Detailed descriptions of the MoCA were provided by Nasreddine et al^4^. The Auditory Verbal Learning Test (AVLT) primarily evaluates subjects' word learning abilities by counting the number of correctly recalled words per trial. Participants initially learn a list of 12 words repeated three times, followed by a short-delay recall after a 5-minute interval. Subsequently, a long-delay recall, cued recall, and recognition test are conducted after a 20-minute interval^5^. These instruments have been validated for cultural and linguistic differences and demonstrated to be reliable and valid within Chinese populations^6-8^.

The TMT comprises two parts. TMT-A involves connecting 25 sequentially numbered circles from 1 to 25. In adapting the test for the Chinese population, TMT-B was modified to include 25 numbers: 13 circles (numbered 1 to 13) and 12 squares (numbered 1 to 12)^9^. The reliability and validity of the Chinese version of the TMT have been confirmed for assessing cognitive impairment in the Chinese population^9^. However, there has been no universally accepted and clearly defined normal range to distinguish the presence of cognitive impairment.

Both the DST and the DSST are derived from the Chinese version of the Wechsler Adult Intelligence Scale-Revised (WAIS-RC)^10^. The DST consists of two tasks: forward and backward digit spans. During the test, the experimenter verbally presents a series of digits at a rate of one digit per second, with the length of the series increasing progressively. Participants must reproduce the digits in the same order for the forward task and in reverse order for the backward task. The score for each task is the total number of correct sequences completed before the participant fails two consecutive trials at a given span length. The final DST score in this study is the sum of the scores from both tasks^11^. Nevertheless, the DST does not provide a clear distinction between cognitive impairment and normal individuals. The DSST involves a digit-symbol encoding table in which each digit from 1 to 9 is paired with a specific symbol. Participants are given 90 seconds to match as many symbols to their corresponding digits as possible on the coding table. Each correctly matched symbol earns one point, with the total possible score ranging from 0 to 90^12^.

In the CDT, participants are instructed as follows: "Please draw a clock, include all the numbers, and set the time to 10 minutes past 11." The scoring criteria for this test were established by Ishiai et al.,^13^. In the CFT, participants are instructed to copy intricate geometric shapes and then reproduce them from memory after a 3-minute interval (CFT-delay). We utilized the Rey-Osterrieth Complex Figure test version, which comprises 18 units, each scored up to two points, yielding a maximum score of 36 points. Osterrieth et al. provided the details on the scoring criteria^14^. Although research on the reliability and validity of the Chinese versions of the CDT and CFT is currently limited, these assessments have been extensively applied in numerous studies involving Chinese populations^15,16^.

In the Verbal Fluency Test (VFT), participants were seated comfortably with their eyes open and were instructed to minimize head movements during the measurement. The task paradigm included a 30-second pre-task baseline period, a 60-second task period, and a 30-second post-task baseline period. The test score is the number of words the participants verbalize. The details of the test in Chinese version are introduced by Quan et al.,^17^.

The Mandarin Chinese versions of the SAS and the SDS were utilized to assess anxiety and depressive symptoms^18-20^. For the SAS, the primary indicator is the total score, which is derived from 20 items, each scored on a scale of up to 4 points. A total score exceeding 40 points indicates anxiety, with higher scores reflecting a greater degree of anxiety. Similarly, the SDS total score serves as the main indicator. The standard score is calculated by multiplying the raw score (the sum of 20 items) by 1.25 and then rounding to the nearest integer. A total score of 50 points or higher suggests depression, with higher scores denoting a more pronounced depressive tendency. All the sensitivity of the measures to detecting cognitive changes we used in the current study have been shown in the Table S21.

**Method S4. Group independent component analysis**

The procedure of ICA involved the following steps: data dimensionally reduction, ICA algorithm application and reconstruction backward to each subject. Firstly, a two-stage principle component analysis (PCA) was conducted to data dimensionally reduction for decreasing computational complexity. Specifically, the preprocessed data for multiple individual was compressed into fractions by the first PCA and then concatenated into a single dataset (i.e., grouped dataset) along the temporal dimension; the group-level dataset was continuously reduced by the second PCA through component dimension. Secondly, independent components (ICs) were calculated by the Infomax algorithm. The data was decomposed into 50 components, which was found to be the optimal number of ICs based on the minimum description length (MDL) criteria^21^. To assess the stability of ICs, this step was repeated 100 times by the ICASSO algorithm. Finally, the ICs for each individual, including time courses and spatial maps, were derived from back reconstruction using group ICA and were conducted Z-scores transform for display. The 13 components were selected from the results of ICA according to the following criteria: (1) IC should exhibited peak activations in grey matter; (2) low spatial overlap with known vascular, ventricular, motion, and susceptibility artifacts and (3) the time courses of the IC dominated by low frequency fluctuations (the ratio of the integral of spectral power below 0.10 Hz to the integral of power between 0.15 and 0.25 Hz)^22^. Through visual inspection, we sorted the 13 ICs into 6 conventional resting-state networks, including default mode network (DMN), central executive network (CEN), salience network (SN), auditory network (AUN), visual network (VN) and sensorimotor network (SMN), referring to the spatial maps and atlas used in previous studies^21,23,24^. Above procedure of independent components extraction and classification are concluded in the followed diagram. In addition, several post-processing steps were performed for noise signal cancelling as follows: linear, quadratic and cubic detrending; multiple regressions of realignment and their temporal derivate; deletion of detected outliers; low-pass filtering at 0.01-0.15 Hz.


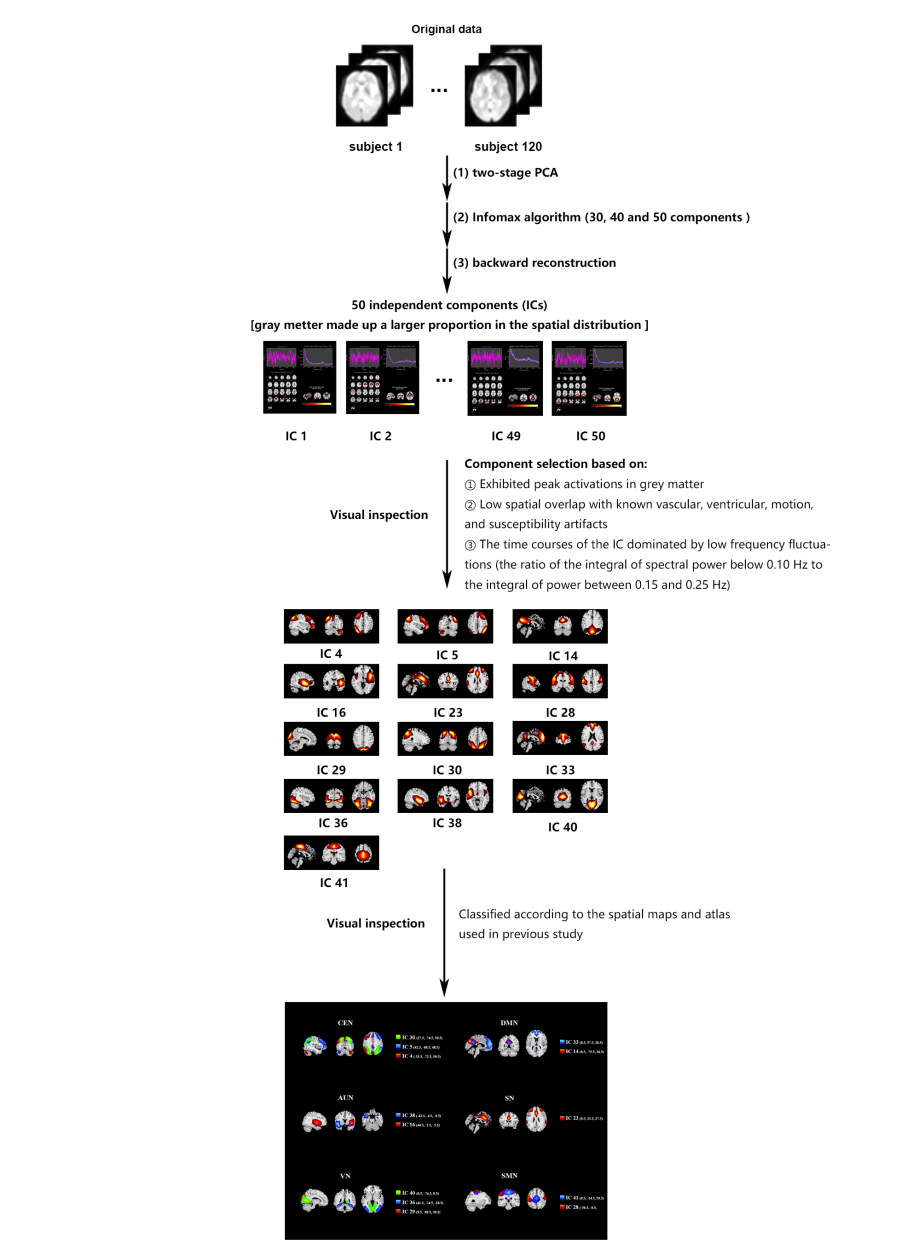


Diagram. The procedure of independent components extraction and classification

**Method S5. Static and Dynamic FNC analysis**

To evaluate changes in FNC between pairs of ICs over time, a sliding-window method was performed to extract FNC signals in a fixed time width from the total time course. This was accomplished using a rectangular window size set to 20 TRs convolved with a Gaussian (σ = 3) and an advancing step of 1 TR^25^. The choice of the window size ensures it is short enough not to suppress the BOLD signal beyond 0.01Hz while also maintains control over false positives in statistical analysis^26^. And step length of a 1 TR is widely used due to be proved for detecting both state changes and durations^27^.

K-mean algorithm was used to automatically evaluate the number of clusters from all the windowed FNC matrices with the square Euclidean distance method after 500 iterations and 150 repeats^28^. The number of clusters was determined by the value of k, varying from 2 to 10, which defined as the ratio of intra- to inter-cluster distances. Based on the elbow method, the optimal number of clusters is inferred as a point on the elbow of curve, representing as the beginning of a small reduction on the within-cluster variability. According to the result of elbow method, the optimal number of clusters was determined to be 6 (k = 6). All the dFNC matrices for each subject were categorized as one of the six clusters based on the similarity with the cluster centroid.

Three temporal properties are defined as follow: fraction time (FT) is defined as the proportion of time spent in each state. Mean dwell time (MDT) represents the average number of contiguous windows belonging to a certain state before transferring to other states. Number of transition (NT) is the average number of state transition among the whole states for all subjects, which reflects the stability of dFNC states for two groups.

**Method S6. Dynamic network topology analysis**

The evaluation of topological organization among the functional connectivity networks across dFNC matrices for each subject was performed on GRETNA software. Using a set of fixed sparsity thresholds^29^, defined as the proportion of the existing number of edges within the maximum possible number of edges, 200 dFNC matrices for each subject were converted to undirected sparse binary matrices, where the value of edge was 1 if the correlations between node i and node j exceeded the predefined thresholds and otherwise was 0. The range of sparsity thresholds was set as 0.2 to 0.4 with an increment of 0.01 based on previous studies while only positive correlations were considered to further analyses.

Referring to previous graph theory-based studies^30^, the area under the curve (AUC) was estimated for global efficiency and local efficiency, which enabled to avert the specific selection of a threshold. Moreover, for the two topological metrics, the AUC in each created sparse matrix was computed, which contained information of the changes in global efficiency and local efficiency across time. Finally, the variance on the AUC of each topological metric over time for each subject was calculated for exploring dynamic characteristics of topological properties in functional connectivity networks.

**Method S7. Multilayer modularity and network switching analysis**

Multilayer network was consisted of dynamic functional network matrices. The detailed procedure of dynamic functional network matrices generation was as follow. Additional preprocessing steps were continuously conducted on the preprocessed data above including temporally detrending with linear order, regressing out nuisance covariates, such as white matter signal, cerebrospinal fluid signal and Friston 24 head motion parameters, and temporally filtering the data between frequencies of 0.01 to 0.1 Hz. The automated anatomical labeling (AAL) 90 atlas was chosen for signal extraction across time series since it defines 90 brain areas into several functional networks, including DMN, CEN, SN, AUN, VN, SMN, cingulo-opercular network (CON), subcortical network (SCN) and none^31,32^. Based on sliding windows method, dynamic functional connectivity (Pearson’s correlation coefficients) matrices between the 90 brain areas of each window were calculated, where the window length was set to 20 TRs (40s) with 1 TRs (2s) step length. Eventually, dynamic functional network matrices (90 × 90 × 201) for each subject were acquired for later multilayer networks analyses.

Using an iterative and ordinal Louvain algorithm, the Multilayer modularity Q was calculated as follows:

$$Q(\gamma,\omega)=\frac{1}{2\mu}\sum_{\mathrm{ijsr}} \left[ \left( A_{\mathrm{ijs}}-\gamma_{s}\frac{k_{\mathrm{is}}k_{\mathrm{js}}}{{2m}_{s}} \right)\delta\left( M_{\mathrm{is}},M_{\mathrm{js}} \right)+\delta\left( i,j \right)\cdot\omega_{\mathrm{jrs}} \right]\delta\left( M_{\mathrm{is}},M_{\mathrm{jr}} \right)$$

Here, μ is the overall edge weight of the network, A_ijs_ is the correlation matrix between node i and j at layer s, k_is_k_js_/2m_s_ describes the matrix with expected connectivity weight under the Newman-Girvan null model. Since only positive matrix values can be calculated in this algorithm, all negative correlations in each layer matrix were excluded. γ_s_ is one of parameter, determining topological structure in the layer s, while ω_jrs_ is another parameter, affecting the topological coupling for node i and j between the adjacent layer r and s. δ is the Kronecker delta, where δ(M_is_, M_js_) and δ(M_is_, M_jr_) are 1 if node i and j belong to the same community and are 0 if they do not belong to the same community. The final output of the algorithm was a 2D matrix (N × W) with integer values representing modules with strong within-network connectivity.

**References:**

1. Szucs D, Ioannidis JP. Sample size evolution in neuroimaging research: An evaluation of highly-cited studies (1990-2012) and of latest practices (2017-2018) in high-impact journals. *Neuroimage*. 2020;221:117164.

2. Geuter S, Qi G, Welsh RC, Wager TD, Lindquist MA. Effect Size and Power in fMRI Group Analysis. *bioRxiv*. 2018:295048.

3. Folstein MF, Folstein SE, McHugh PR. "Mini-mental state". A practical method for grading the cognitive state of patients for the clinician. *J Psychiatr Res*. 1975;12(3):189-98.

4. Nasreddine ZS, Phillips NA, Bédirian V, et al. The Montreal Cognitive Assessment, MoCA: a brief screening tool for mild cognitive impairment. *J Am Geriatr Soc*. 2005;53(4):695-9.

5. Zhao Q, Lv Y, Zhou Y, Hong Z, Guo Q. Short-term delayed recall of auditory verbal learning test is equivalent to long-term delayed recall for identifying amnestic mild cognitive impairment. *PLoS One*. 2012;7(12):e51157. doi:10.1371/journal.pone.0051157

6. Guo QH, Lv CZ, Hong Z. Auditory verbal memory test in Chinese elderly. *Chinese Mental Health Journal*. 2001;15:13-15.

7. Katzman R, Zhang MY, Ouang Ya Q, et al. A Chinese version of the Mini-Mental State Examination; impact of illiteracy in a Shanghai dementia survey. *J Clin Epidemiol*. 1988;41(10):971-8.

8. Lu J, Li D, Li F, et al. Montreal cognitive assessment in detecting cognitive impairment in Chinese elderly individuals: a population-based study. *J Geriatr Psychiatry Neurol*. 2011;24(4):184-90.

9. Wei M, Shi J, Li T, et al. Diagnostic Accuracy of the Chinese Version of the Trail-Making Test for Screening Cognitive Impairment. *J Am Geriatr Soc*. 2018;66(1):92-99.

10. Dai XY, Ryan JJ, Paolo AM, Harrington RG. Factor analysis of the mainland Chinese version of the Wechsler Adult Intelligence Scale (WAIS-RC) in a brain-damaged sample. *Int J Neurosci*. 1990;55(2-4):107-11.

11. Hester RL, Kinsella GJ, Ong B. Effect of age on forward and backward span tasks. *J Int Neuropsychol Soc*. 2004;10(4):475-81.

12. Lu Z, He R, Zhang Y, et al. Relationship between Whole-Blood Magnesium and Cognitive Performance among Chinese Adults. *Nutrients*. 2023;15(12):2706

13. Ishiai S, Sugishita M, Ichikawa T, Gono S, Watabiki S. Clock-drawing test and unilateral spatial neglect. *Neurology*. 1993;43(1):106-10.

14. Osterrieth PA. Le test de copie d'une figure complexe; contribution à l'étude de la perception et de la mémoire. [Test of copying a complex figure; contribution to the study of perception and memory.]. *Archives de Psychologie*. 1944;30:206-356.

15. Guo Z, Xu G, Wang R, et al. Free thyroxine, brain frailty and clock drawing test performance in patients with acute minor stroke or transient ischaemic attack. *Clin Endocrinol (Oxf)*. 2022;96(2):175-183.

16. Tang WK, Lau CG, Ungvari GS, Lin SK, Lane HY. Recovery of cognitive functioning following abstinence from ketamine. *Addict Behav*. 2019;99:106081.

17. Quan W, Wu T, Li Z, Wang Y, Dong W, Lv B. Reduced prefrontal activation during a verbal fluency task in Chinese-speaking patients with schizophrenia as measured by near-infrared spectroscopy. *Prog Neuropsychopharmacol Biol Psychiatry*. 2015;58:51-8.

18. Zhang Q, Li F, Zhang H, Yu X, Cong Y. Effects of nurse-led home-based exercise & cognitive behavioral therapy on reducing cancer-related fatigue in patients with ovarian cancer during and after chemotherapy: A randomized controlled trial. *Int J Nurs Stud*. 2018;78:52-60.

19. Yin X, Gou M, Xu J, et al. Efficacy and safety of acupuncture treatment on primary insomnia: a randomized controlled trial. *Sleep Med*. 2017;37:193-200.

20. Zung WW. A rating instrument for anxiety disorders. *Psychosomatics*. 1971;12(6):371-9.

21. Lan L, Liu Y, Wu Y, et al. Specific brain network predictors of interventions with different mechanisms for tinnitus patients. *EBioMedicine*. 2022;76:103862.

22. Cordes D, Haughton VM, Arfanakis K, et al. Mapping functionally related regions of brain with functional connectivity MR imaging. *AJNR Am J Neuroradiol*. 2000;21(9):1636-44.

23. Xing C, Chen YC, Shang S, et al. Abnormal Static and Dynamic Functional Network Connectivity in Patients With Presbycusis. *Front Aging Neurosci*. 2021;13:774901.

24. Li F, Lu L, Shang S, et al. Altered static and dynamic functional network connectivity in post-traumatic headache. *J Headache Pain*. 2021;22(1):137.

25. Zhang J, Cui H, Yang H, et al. Dynamic changes of large-scale resting-state functional networks in major depressive disorder. *Prog Neuropsychopharmacol Biol Psychiatry*. 2021;111:110369.

26. Zalesky A, Breakspear M. Towards a statistical test for functional connectivity dynamics. *Neuroimage*. 2015;114:466-70.

27. Shakil S, Lee CH, Keilholz SD. Evaluation of sliding window correlation performance for characterizing dynamic functional connectivity and brain states. *Neuroimage*. 2016;133:111-128.

28. Malhi GS, Das P, Outhred T, Bryant RA, Calhoun V. Resting-state neural network disturbances that underpin the emergence of emotional symptoms in adolescent girls: resting-state fMRI study. *Br J Psychiatry*. 2019;215(3):545-551.

29. Achard S, Bullmore E. Efficiency and cost of economical brain functional networks. *PLoS Comput Biol*. 2007;3(2):e17.

30. Yu Q, Erhardt EB, Sui J, et al. Assessing dynamic brain graphs of time-varying connectivity in fMRI data: application to healthy controls and patients with schizophrenia. *Neuroimage*. 2015;107:345-355.

31. Power JD, Cohen AL, Nelson SM, et al. Functional network organization of the human brain. *Neuron*. 2011;72(4):665-78.

32. Xu Y, Shang H, Lu H, Zhang J, Yao L, Long Z. Altered Dynamic Functional Connectivity in Subcortical Ischemic Vascular Disease With Cognitive Impairment. *Front Aging Neurosci*. 2021;13:758137.

33. Tsoi KK, Chan JY, Hirai HW, Wong SY, Kwok TC. Cognitive Tests to Detect Dementia: A Systematic Review and Meta-analysis. *JAMA Intern Med*. 2015;175(9):1450-8.

34. Zhao Q, Lv Y, Zhou Y, Hong Z, Guo Q. Short-term delayed recall of auditory verbal learning test is equivalent to long-term delayed recall for identifying amnestic mild cognitive impairment. *PLoS One*. 2012;7(12):e51157.

35. Hester RL, Kinsella GJ, Ong B. Effect of age on forward and backward span tasks. *J Int Neuropsychol Soc*. 2004;10(4):475-81.

36. Leung JL, Lee GT, Lam YH, Chan RC, Wu JY. The use of the Digit Span Test in screening for cognitive impairment in acute medical inpatients. *Int Psychogeriatr*. 2011;23(10):1569-74.

37. O'Sullivan M, Morris RG, Markus HS. Brief cognitive assessment for patients with cerebral small vessel disease. *J Neurol Neurosurg Psychiatry*. 2005;76(8):1140-5.

38. Park J, Jeong E, Seomun G. The clock drawing test: A systematic review and meta-analysis of diagnostic accuracy. *J Adv Nurs*. 2018;74(12):2742-2754.

39. Tsatali M, Avdikou K, Gialaouzidis M, et al. The discriminant validity of Rey Complex Figure Test (RCFT) in subjective cognitive decline, mild cognitive impairment (multiple domain) and Alzheimer's disease dementia (ADD; mild stage) in Greek older adults. *Appl Neuropsychol Adult*. 2022:1-10.

40. Chiu HF, Chan CK, Lam LC, et al. The modified Fuld Verbal Fluency Test: a validation study in Hong Kong. *J Gerontol B Psychol Sci Soc Sci*. 1997;52(5):P247-50.


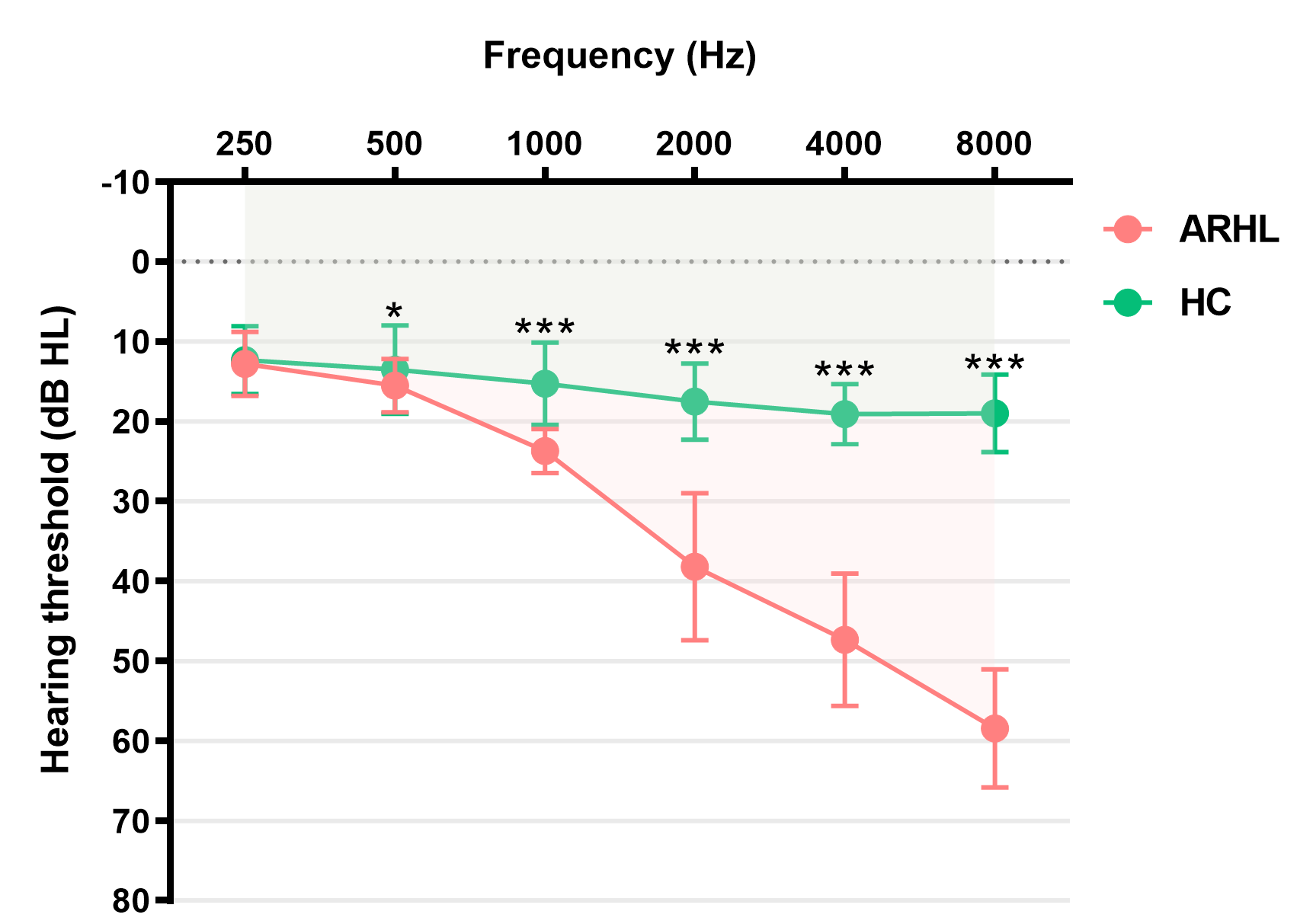


**Fig. S1.** Mean hearing thresholds (+/- 1 standard deviation) at 0.5, 1, 2, 4, 8 kHz were significantly higher in age-related hearing loss (ARHL) group than in healthy controls (HC) (^*^*p* < 0.05, ^***^*p* < 0.001). HC: healthy controls; ARHL: age-related hearing loss.


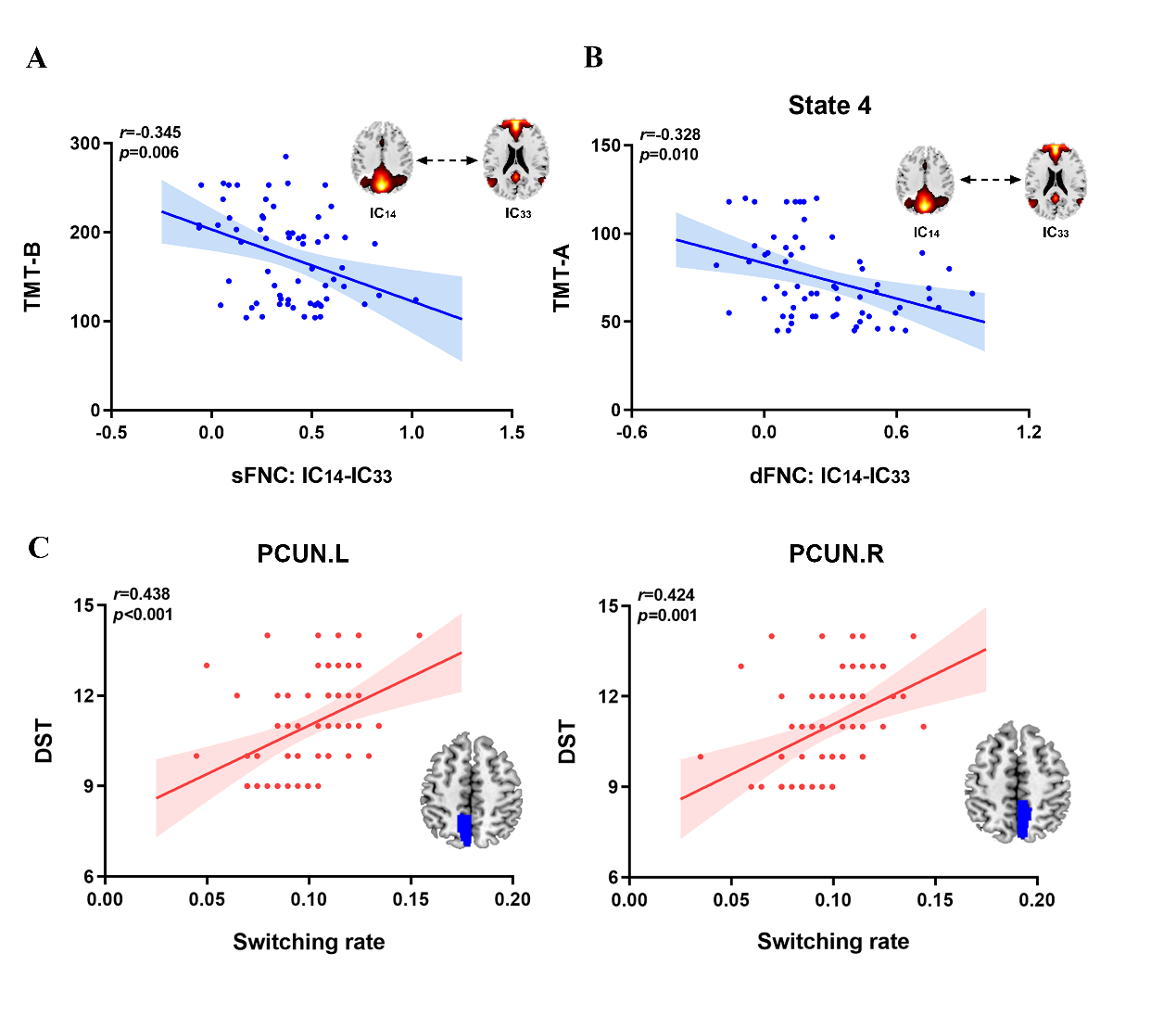


**Fig. S2.** Correlations between clinical traits and network metrics. (A) Negative correlation between functional network connectivity (IC_14_-IC_33_) and TMT-B scores for ARHL group controlled for age, gender and educational level (*r*=-0.345, *p*=0.006, Spearman’s correlation). Each dot denotes an individual-level value. (B) Negative correlation between dynamic functional network connectivity (IC_14_-IC_33_) in state 4 and TMT-A scores for ARHL group, controlled for age, gender and educational level (*r*=-0.328, *p*=0.010, Spearman correlation). Each dot denotes an individual-level value. (C) Positive correlation between network switching rate in bilateral precuneus and DST for ARHL group, controlled for age, gender and educational level (*r_L_*=0.438, *p_L_*<0.001; *r_R_*=0.424, *p_R_*<0.001, Spearman’s correlation). Each dot denotes an individual-level value. The shaded areas indicates 95% CIs. IC: independent component; sFNC: static functional network connectivity; TMT-B: Trail Making Test-Part B; TMT-A: Trail Making Test-Part A; DST: Digit Span Test; PCUN.L: left precuneus; PCUN.R: right precuneus.

**Table S1.** Between-group comparisons for node switching rate in different functional networks

| Functional network | Node | *t* | *p* | *q_FDR_* |
| --- | --- | --- | --- | --- |
| ARHL vs. HC |  |  |  |  |
| Default mode network | SFGmed.R | -2.966 | 0.004 | **0.030^*^** |
|  | PCUN.L | -2.815 | 0.006 | **0.040^*^** |
|  | PCUN.R | -3.364 | 0.001 | **0.016^*^** |
| Central executive network | SFGdor.R | -3.919 | <0.001 | **0.007^**^** |
|  | MFG.R | -3.712 | <0.001 | **0.007^**^** |
|  | ORBmid.L | -3.383 | <0.001 | **0.016^*^** |
| Salience network | IFGtriang.L | -3.318 | 0.001 | **0.016^*^** |
|  | INS.R | -3.042 | 0.003 | **0.029^*^** |
|  | DCG.L | -2.984 | 0.003 | **0.030^*^** |
| Auditory network | ROL.R | -2.692 | 0.008 | **0.049^*^** |
|  | SMG.R | -3.188 | 0.002 | **0.021^*^** |
| Subcortical network | CAU.R | -3.729 | <0.001 | **0.007^**^** |
| Visual network | FFG.L | -2.855 | 0.005 | **0.038^*^** |
| Sensorimotor network | SMA.R | -3.780 | <0.001 | **0.007^**^** |

General linear model was applied for comparing the differences of node switching rate in functional networks between ARHL and HC group, controlled for age, gender and educational level, ^*^*q_FDR_* < 0.05, ^**^ *q_FDR_* < 0.01; FDR: false discovery rate; ARHL: age-related hearing loss; HC: healthy control; ORBmid.L: left orbital middle frontal gyrus; IFGtriang.L: left triangular inferior frontal gyrus; DCG.L: median cingulate and paracingulate gyrus; PCUN.L: left precuneus; FFG.L: left fusiform gyrus; SFGmed.R: right medial superior frontal gyrus; SFGdor.R: right dorsolateral superior frontal gyrus; SMA.R: right supplementary motor area; MFG.R: right middle frontal gyrus; CAU.R: right caudate nucleus; INS.R: right insula; ROL.R: right rolandic operculum; SMG.R: right supramarginal gyrus; PCUN.R: right precuneus.

**Table S2.** Validation analysis for significant differences on static FNC between ARHL patients and healthy controls after controlling for the mean FD

| sFNC | *t* | *p* | *q_FDR_* |
| --- | --- | --- | --- |
| ARHL vs. HC |  |  |  |
| CEN(IC_30_)-DMN(IC_33_) | 3.245 | 0.002 | **0.030^*^** |
| DMN(IC_14_)-DMN(IC_33_) | -3.303 | 0.002 | **0.030^*^** |
| DMN(IC_14_)-VN(IC_40_) | 4.083 | <0.001 | **0.007^**^** |
| SN(IC_23_)- DMN(IC_33_) | 3.380 | <0.001 | **0.030^*^** |

General linear model was applied for comparing the differences on static FNC between ARHL and HC group, controlled for age, gender, educational level and the mean FD, ^*^*q_FDR_* < 0.05, ^**^ *q_FDR_* < 0.01; FDR: false discovery rate; ARHL: age-related hearing loss; HC: healthy control; IC: independent component; DMN: default mode network; CEN: central executive network; SN: salience network; VN: visual network.

**Table S3.** Validation analysis for significant differences on dynamic FNC between ARHL patients and healthy controls after controlling for the mean FD

| states | sFNC | *t* | *p* | *q_FDR_* |
| --- | --- | --- | --- | --- |
| ARHL vs. HC |  |  |  |  |
| State 1 | CEN(IC_30_)-VN(IC_40_) | 4.058 | <0.001 | **0.011^*^** |
|  | DMN(IC_14_)-DMN(IC_33_) | -3.576 | <0.001 | **0.027^*^** |
| State 4 | DMN(IC_14_)-DMN(IC_33_) | -4.174 | <0.001 | **0.005^**^** |
|  | SN(IC_23_)- DMN(IC_33_) | 3.760 | <0.001 | **0.010^*^** |
|  | VN(IC_29_)- CEN(IC_30_) | 3.328 | 0.001 | **0.030^*^** |

General linear model was applied for comparing the differences on static FNC between ARHL and HC group, controlled for age, gender, educational level and the mean FD, ^*^*q_FDR_* < 0.05, ^**^ *q_FDR_* < 0.01; FDR: false discovery rate; ARHL: age-related hearing loss; HC: healthy control; IC: independent component; DMN: default mode network; CEN: central executive network; SN: salience network; VN: visual network.

**Table S4.** Validation analysis for significant differences on network switching rate between ARHL patients and healthy controls after controlling for the mean FD

| Functional network | Node | *t* | *p* | *q_FDR_* |
| --- | --- | --- | --- | --- |
| ARHL vs. HC |  |  |  |  |
| Default mode network | SFGmed.R | -3.070 | 0.003 | **0.027^*^** |
|  | PCUN.R | -3.226 | 0.002 | **0.021^*^** |
| Central executive network | SFGdor.R | -3.820 | <0.001 | **0.012^*^** |
|  | MFG.R | -3.518 | <0.001 | **0.013^*^** |
|  | ORBmid.L | -3.480 | <0.001 | **0.013^*^** |
| Salience network | IFGtriang.L | -3.241 | 0.002 | **0.021^*^** |
|  | INS.R | -3.093 | 0.003 | **0.027^*^** |
|  | DCG.L | -2.841 | 0.005 | **0.044^*^** |
| Auditory network | SMG.R | -3.000 | 0.003 | **0.030^*^** |
| Subcortical network | CAU.R | -3.547 | <0.001 | **0.013^**^** |
| Visual network | FFG.L | -2.767 | 0.007 | **0.049^*^** |
| Sensorimotor network | SMA.R | -3.766 | <0.001 | **0.012^**^** |

General linear model was applied for comparing the differences of node switching rate in functional networks between ARHL and HC group, controlled for age, gender, educational level and the mean FD, ^*^*q_FDR_* < 0.05, ^**^ *q_FDR_* < 0.01; FDR: false discovery rate; ARHL: age-related hearing loss; HC: healthy control; ORBmid.L: left orbital middle frontal gyrus; IFGtriang.L: left triangular inferior frontal gyrus; DCG.L: median cingulate and paracingulate gyrus; PCUN.L: left precuneus; FFG.L: left fusiform gyrus; SFGmed.R: right medial superior frontal gyrus; SFGdor.R: right dorsolateral superior frontal gyrus; SMA.R: right supplementary motor area; MFG.R: right middle frontal gyrus; CAU.R: right caudate nucleus; INS.R: right insula; SMG.R: right supramarginal gyrus; PCUN.R: right precuneus; DMN: default mode network; CEN: central executive network; SN: salience network; VN: visual network; SMN: sensorimotor network; AUN: auditory network; SCN: subcortical network.

**Table S5.** Correlations between the severity of hearing loss and abnormal static functional network connectivity in individuals with ARHL

| Severity of hearing loss | sFNC | *r* | *p* | *q_FDR_* |
| --- | --- | --- | --- | --- |
| Mean hearing thresholds | CEN(IC_30_)-DMN(IC_33_) | 0.137 | 0.285 | 0.380 |
|  | DMN(IC_14_)-DMN(IC_33_) | 0.197 | 0.123 | 0.380 |
|  | DMN(IC_14_)-VN(IC_40_) | 0.005 | 0.970 | 0.970 |
|  | SN(IC_23_)- DMN(IC_33_) | 0.162 | 0.206 | 0.380 |

Partial correlation analysis was applied for evaluating the correlation between the severity of hearing loss and abnormal static functional network connectivity in ARHL, controlled for age, gender and educational level, FDR: false discovery rate; sFNC: static functional network connectivity; IC: independent component; DMN: default model network; SN: salience network; VN: visual network; CEN: central executive network.

**Table S6.** Correlations between the severity of hearing loss and abnormal dynamic metrics in individuals with ARHL

| Severity of hearing loss | Abnormal dynamic metrics | *r* | *p* | *q_FDR_* |
| --- | --- | --- | --- | --- |
| Mean hearing thresholds | Fraction time in state 3 | 0.042 | 0.746 | - |
|  | Number of transitions | 0.149 | 0.242 | - |

Partial correlation analysis was applied for evaluating the correlation between the severity of hearing loss and abnormal dynamic metrics in individuals with ARHL, controlled for age, gender and educational level, FDR: false discovery rate.

**Table S7.** Correlations between the severity of hearing loss and abnormal dynamic functional network connectivity in state 4 in individuals with ARHL

| Severity of hearing loss | dFNC | *r* | *p* | *q_FDR_* |
| --- | --- | --- | --- | --- |
| Mean hearing thresholds | VN(IC_29_)-CEN(IC_30_) | 0.003 | 0.986 | 0.986 |
|  | DMN(IC_14_)-DMN(IC_33_) | 0.240 | 0.201 | 0.335 |
|  | DMN(IC_14_)-VN(IC_40_) | -0.248 | 0.186 | 0.335 |
|  | SN(IC_23_)-DMN(IC_33_) | 0.043 | 0.822 | 0.986 |
|  | VN(IC_36_)-VN(IC_40_) | -0.304 | 0.103 | 0.335 |

Partial correlation analysis was applied for evaluating the correlation between the severity of hearing loss and abnormal dynamic functional network connectivity in state 4 in individuals with ARHL, controlled for age, gender and educational level, FDR: false discovery rate. dFNC: dynamic functional network connectivity; IC: independent component; DMN: default model network; SN: salience network; CEN: central executive network; VN: visual network; AUN: auditory network; SMN: sensory motor network.

**Table S8.** Correlations between the severity of hearing loss and abnormal dynamic functional network connectivity variability in individuals with ARHL

| Severity of hearing loss | dFNC variability | *r* | *p* | *q_FDR_* |
| --- | --- | --- | --- | --- |
| Mean hearing thresholds | DMN(IC_14_)-AUN(IC_38_) | 0.010 | 0.937 | 0.937 |
|  | AUN(IC_16_)-SN(IC_23_) | 0.094 | 0.462 | 0.924 |

Partial correlation analysis was applied for evaluating the correlation between the severity of hearing loss and abnormal dynamic functional network connectivity variability in individuals with ARHL, controlled for age, gender and educational level, FDR: false discovery rate. dFNC: dynamic functional network connectivity; IC: independent component; DMN: default model network; SN: salience network; AUN: auditory network.

**Table S9.** Correlation within aberrant sFNC in patients with ARHL

| sFNC | sFNC | *r* | *p* | *q_FDR_* |
| --- | --- | --- | --- | --- |
| CEN(IC_30_)-DMN(IC_33_) | DMN(IC_14_)-DMN(IC_33_) | -0.167^a^ | 0.190 | 0.286 |
|  | DMN(IC_14_)-VN(IC_40_) | 0.376^a^ | 0.002 | **0.005^***^** |
|  | SN(IC_23_)-DMN(IC_33_) | 0.696^a^ | <0.001 | **<0.001^***^** |
| DMN(IC_14_)-DMN(IC_33_) | DMN(IC_14_)- VN(IC_40_) | 0.007^a^ | 0.957 | 0.957 |
|  | SN(IC_23_)- DMN(IC_33_) | -0.143^a^ | 0.263 | 0.315 |
| DMN(IC_14_)-VN(IC_40_) | SN(IC_23_)- DMN(IC_33_) | 0.434^a^ | <0.001 | **0.001^***^** |

Partial correlation analysis was applied for evaluating the correlation between different sFNC, controlled for age, gender and educational level, ^***^*q_FDR_* < 0.005; FDR: false discovery rate; ^a^: Pearson’s correlation; sFNC, static functional network connectivity; IC: independent component; DMN: default model network; SN: salience network; VN: visual network.

**Table S10.** Correlation between abnormal sFNC and fraction time in state 3 in patients with ARHL

| Fraction time | sFNC | *r* | *p* | *q_FDR_* |
| --- | --- | --- | --- | --- |
| Fraction time in state 3 | CEN(IC_30_)-DMN(IC_33_) | -0.583^b^ | <0.001 | **<0.001^***^** |
|  | DMN(IC_14_)-DMN(IC_33_) | 0.297^b^ | 0.018 | **0.018^*^** |
|  | DMN(IC_14_)-VN(IC_40_) | -0.352^b^ | 0.005 | **0.006^**^** |
|  | SN(IC_23_)- DMN(IC_33_) | -0.655^b^ | <0.001 | **<0.001^***^** |

Partial correlation analysis was applied for evaluating the correlation between sFNC and fraction time, controlled for age, gender and educational level, ^*^*q_FDR_* < 0.05, ^**^ *q_FDR_* < 0.01, ^***^ *q_FDR_* < 0.005; FDR: false discovery rate; ^b^: Spearman’s correlation; sFNC, static functional network connectivity; IC: independent component; DMN: default model network; SN: salience network; VN: visual network; CEN: central executive network.

**Table S11.** Correlation between aberrant network switching rate and fraction time in state 3 in patients with ARHL

| Fraction time | Functional network | Network switching rate | *r* | *p* | *q_FDR_* |
| --- | --- | --- | --- | --- | --- |
| Fraction time in state 3 | DMN | Frontal_Sup_Medial_R | 0.076^b^ | 0.551 | 0.648 |
|  |  | Precuneus_L | 0.314^b^ | 0.012 | 0.057 |
|  |  | Precuneus_R | 0.318^b^ | 0.011 | 0.057 |
|  | CEN | Frontal_Sup_R | 0.219^b^ | 0.085 | 0.198 |
|  |  | Frontal_Mid_R | 0.128^b^ | 0.316 | 0.491 |
|  |  | Frontal_Mid_Orb_L | 0.229^b^ | 0.071 | 0.198 |
|  | SN | Frontal_Inf_Tri_L | 0.138^b^ | 0.282 | 0.491 |
|  |  | Insula_R | -0.037^b^ | 0.773 | 0.821 |
|  |  | Cingulum_Mid_L | 0.183^b^ | 0.151 | 0.301 |
|  | AUN | Rolandic_Oper_R | -0.029^b^ | 0.821 | 0.821 |
|  |  | SupraMarginal_R | 0.104^b^ | 0.417 | 0.584 |
|  | SMN | Supp_Motor_Area_R | 0.352^b^ | 0.005 | 0.057 |
|  | SCN | Caudate_R | 0.076^b^ | 0.555 | 0.648 |
|  | VN | Fusiform_L | 0.253^b^ | 0.046 | 0.160 |

Partial correlation analysis was applied for evaluating the correlation between fraction time in state 3 and network switching rate in several networks, controlled for age, gender and educational level; FDR: false discovery rate; ^b^: Spearman’s correlation; dFNC, dynamic functional network connectivity; IC: independent component; DMN: default model network; CEN: central executive network; SN: salience network; AUN: auditory network; SMN: sensory motor network; SCN: subcortical network; VN: visual network; Frontal_Sup_Medial_R: right superior medial frontal gyrus; Precuneus_L: left precuneus; Precuneus_R: right precuneus; Frontal_Sup_R: right dorsolateral superior frontal gyrus; Frontal_Mid_R: right middle frontal gyrus; Frontal_Mid_Orb_L: left orbital middle frontal gyrus; Frontal_Inf_Tri_L: left triangular inferior frontal gyrus; Insula_R: right insula; Cingulum_Mid_L: left median cingulate gyrus; Rolandic_Oper_R: right rolandic operculum; SupraMarginal_R: right supramarginal gyrus; Supp_Motor_Area_R: right supplementary motor area; Caudate_R: right caudate nucleus; Fusiform_L: left fusiform gyrus.

**Table S12.** Correlation between aberrant sFNC and abnormal network switching rate in DMN in patients with ARHL

| sFNC | Functional network | Network switching rate | *r* | *p* | *q_FDR_* |
| --- | --- | --- | --- | --- | --- |
| CEN(IC_30_)-DMN(IC_33_) | DMN | Frontal_Sup_Medial_R | 0.089^a^ | 0.488 | 0.780 |
|  |  | Precuneus_L | 0.238^a^ | 0.061 | 0.373 |
|  |  | Precuneus_R | 0.209^a^ | 0.101 | 0.413 |
|  | CEN | Frontal_Sup_R | 0.168^b^ | 0.187 | 0.498 |
|  |  | Frontal_Mid_R | 0.112^a^ | 0.384 | 0.716 |
|  |  | Frontal_Mid_Orb_L | 0.190^a^ | 0.136 | 0.414 |
|  | SN | Frontal_Inf_Tri_L | 0.196^a^ | 0.123 | 0.414 |
|  |  | Insula_R | 0.010^a^ | 0.935 | 0.970 |
|  |  | Cingulum_Mid_L | 0.218^a^ | 0.086 | 0.414 |
|  | AUN | Rolandic_Oper_R | 0.012^a^ | 0.923 | 0.970 |
|  |  | SupraMarginal_R | 0.052^b^ | 0.685 | 0.844 |
|  | SMN | Supp_Motor_Area_R | 0.396^a^ | 0.001 | **0.024^*^** |
|  | SCN | Caudate_R | 0.186^a^ | 0.145 | 0.414 |
|  | VN | Fusiform_L | 0.091^a^ | 0.478 | 0.780 |
| DMN(IC_14_)-DMN(IC_33_) | DMN | Frontal_Sup_Medial_R | 0.118^a^ | 0.358 | 0.691 |
|  |  | Precuneus_L | 0.031^a^ | 0.810 | 0.890 |
|  |  | Precuneus_R | 0.001^a^ | 0.991 | 0.991 |
|  | CEN | Frontal_Sup_R | 0.127^b^ | 0.321 | 0.675 |
|  |  | Frontal_Mid_R | 0.042^a^ | 0.741 | 0.844 |
|  |  | Frontal_Mid_Orb_L | 0.050^a^ | 0.698 | 0.844 |
|  | SN | Frontal_Inf_Tri_L | 0.134^a^ | 0.294 | 0.675 |
|  |  | Insula_R | 0.058^a^ | 0.651 | 0.844 |
|  |  | Cingulum_Mid_L | 0.054^a^ | 0.674 | 0.844 |
|  | AUN | Rolandic_Oper_R | 0.082^a^ | 0.521 | 0.794 |
|  |  | SupraMarginal_R | 0.126^b^ | 0.326 | 0.675 |
|  | SMN | Supp_Motor_Area_R | 0.006^a^ | 0.963 | 0.980 |
|  | SCN | Caudate_R | 0.056^a^ | 0.661 | 0.844 |
|  | VN | Fusiform_L | 0.082^a^ | 0.525 | 0.794 |
| DMN(IC_14_)-VN(IC_40_) | DMN | Frontal_Sup_Medial_R | 0.041^a^ | 0.752 | 0.844 |
|  |  | Precuneus_L | 0.403^a^ | 0.001 | **0.024^*^** |
|  |  | Precuneus_R | 0.303^a^ | 0.016 | 0.147 |
|  | CEN | Frontal_Sup_R | 0.047^b^ | 0.714 | 0.844 |
|  |  | Frontal_Mid_R | 0.105^a^ | 0.415 | 0.749 |
|  |  | Frontal_Mid_Orb_L | 0.184^a^ | 0.148 | 0.414 |
|  | SN | Frontal_Inf_Tri_L | 0.092^a^ | 0.475 | 0.780 |
|  |  | Insula_R | 0.189^a^ | 0.137 | 0.414 |
|  |  | Cingulum_Mid_L | 0.200^a^ | 0.116 | 0.414 |
|  | AUN | Rolandic_Oper_R | 0.060^a^ | 0.638 | 0.844 |
|  |  | SupraMarginal_R | 0.040^b^ | 0.753 | 0.844 |
|  | SMN | Supp_Motor_Area_R | 0.295^a^ | 0.019 | 0.152 |
|  | SCN | Caudate_R | 0.051^a^ | 0.690 | 0.844 |
|  | VN | Fusiform_L | 0.337^a^ | 0.007 | 0.936 |
| SN(IC_23_)- DMN(IC_33_) | DMN | Frontal_Sup_Medial_R | 0.157^a^ | 0.218 | 0.555 |
|  |  | Precuneus_L | 0.334^a^ | 0.007 | 0.086 |
|  |  | Precuneus_R | 0.333^a^ | 0.008 | 0.086 |
|  | CEN | Frontal_Sup_R | 0.273^b^ | 0.031 | 0.214 |
|  |  | Frontal_Mid_R | 0.212^a^ | 0.096 | 0.414 |
|  |  | Frontal_Mid_Orb_L | 0.131^a^ | 0.307 | 0.675 |
|  | SN | Frontal_Inf_Tri_L | 0.233^a^ | 0.067 | 0.373 |
|  |  | Insula_R | 0.101^a^ | 0.433 | 0.758 |
|  |  | Cingulum_Mid_L | 0.119^a^ | 0.354 | 0.691 |
|  | AUN | Rolandic_Oper_R | 0.069^a^ | 0.590 | 0.844 |
|  |  | SupraMarginal_R | 0.055^b^ | 0.669 | 0.844 |
|  | SMN | Supp_Motor_Area_R | 0.462^a^ | <0.001 | **0.008^**^** |
|  | SCN | Caudate_R | 0.187^a^ | 0.142 | 0.414 |
|  | VN | Fusiform_L | 0.132^a^ | 0.304 | 0.675 |

Partial correlation analysis was applied for evaluating the correlation between sFNC and network switching rate, controlled for age, gender and educational level, ^*^*q_FDR_* < 0.05; ^**^*q_FDR_* < 0.01; FDR: false discovery rate; ^a^: Pearson’s correlation; ^b^: Spearman’s correlation; sFNC, static functional network connectivity; IC: independent component; DMN: default model network; CEN: central executive network; SN: salience network; AUN: auditory network; SMN: sensory motor network; SCN: subcortical network; VN: visual network; Frontal_Sup_Medial_R: right superior medial frontal gyrus; Precuneus_L: left precuneus; Precuneus_R: right precuneus; Frontal_Sup_R: right dorsolateral superior frontal gyrus; Frontal_Mid_R: right middle frontal gyrus; Frontal_Mid_Orb_L: left orbital middle frontal gyrus; Frontal_Inf_Tri_L: left triangular inferior frontal gyrus; Insula_R: right insula; Cingulum_Mid_L: left median cingulate gyrus; Rolandic_Oper_R: right rolandic operculum; SupraMarginal_R: right supramarginal gyrus; Supp_Motor_Area_R: right supplementary motor area; Caudate_R: right caudate nucleus; Fusiform_L: left fusiform gyrus.

**Table S13.** Correlation between aberrant dFNC variability and abnormal network switching rate in patients with ARHL

| dFNC variability | Functional network | Network switching rate | *r* | *p* | *q_FDR_* |
| --- | --- | --- | --- | --- | --- |
| DMN(IC_14_)-AUN(IC_38_) | DMN | Frontal_Sup_Medial_R | -0.230^a^ | 0.070 | 0.279 |
|  |  | Precuneus_L | -0.284^a^ | 0.024 | 0.145 |
|  |  | Precuneus_R | -0.405^a^ | 0.001 | 0.014^*^ |
|  | CEN | Frontal_Sup_R | -0.135^b^ | 0.291 | 0.508 |
|  |  | Frontal_Mid_R | -0.443^a^ | <0.001 | **0.008^**^** |
|  |  | Frontal_Mid_Orb_L | -0.281^a^ | 0.026 | 0.145 |
|  | SN | Frontal_Inf_Tri_L | -0.341^a^ | 0.006 | 0.059 |
|  |  | Insula_R | -0.054^a^ | 0.672 | 0.742 |
|  |  | Cingulum_Mid_L | -0.082^a^ | 0.525 | 0.652 |
|  | AUN | Rolandic_Oper_R | -0.056^a^ | 0.664 | 0.742 |
|  |  | SupraMarginal_R | -0.086^b^ | 0.501 | 0.652 |
|  | SMN | Supp_Motor_Area_R | -0.197^a^ | 0.122 | 0.283 |
|  | SCN | Caudate_R | -0.013^a^ | 0.922 | 0.956 |
|  | VN | Fusiform_L | 0.006^a^ | 0.963 | 0.963 |
| AUN(IC_16_)-SN(IC_23_) | DMN | Frontal_Sup_Medial_R | -0.202^a^ | 0.113 | 0.283 |
|  |  | Precuneus_L | -0.187^a^ | 0.141 | 0.283 |
|  |  | Precuneus_R | -0.193^a^ | 0.130 | 0.283 |
|  | CEN | Frontal_Sup_R | 0.103^b^ | 0.422 | 0.594 |
|  |  | Frontal_Mid_R | -0.079^a^ | 0.536 | 0.652 |
|  |  | Frontal_Mid_Orb_L | -0.259^a^ | 0.040^*^ | 0.187 |
|  | SN | Frontal_Inf_Tri_L | -0.192^a^ | 0.132 | 0.283 |
|  |  | Insula_R | -0.051^a^ | 0.689 | 0.742 |
|  |  | Cingulum_Mid_L | -0.205^a^ | 0.106 | 0.283 |
|  | AUN | Rolandic_Oper_R | -0.153^a^ | 0.230 | 0.429 |
|  |  | SupraMarginal_R | -0.103^b^ | 0.424 | 0.594 |
|  | SMN | Supp_Motor_Area_R | -0.188^a^ | 0.141 | 0.283 |
|  | SCN | Caudate_R | -0.108^a^ | 0.399 | 0.594 |
|  | VN | Fusiform_L | -0.104^a^ | 0.418 | 0.594 |

Partial correlation analysis was applied for evaluating the correlation between dFNC variability and network switching rate, controlled for age, gender and educational level, ^*^*q_FDR_* < 0.05, ^**^*q_FDR_* < 0.01; FDR: false discovery rate; ^a^: Pearson’s correlation; ^b^: Spearman’s correlation; dFNC, dynamic functional network connectivity; IC: independent component; DMN: default model network; CEN: central executive network; SN: salience network; AUN: auditory network; SMN: sensory motor network; SCN: subcortical network; VN: visual network; Frontal_Sup_Medial_R: right superior medial frontal gyrus; Precuneus_L: left precuneus; Precuneus_R: right precuneus; Frontal_Sup_R: right dorsolateral superior frontal gyrus; Frontal_Mid_R: right middle frontal gyrus; Frontal_Mid_Orb_L: left orbital middle frontal gyrus; Frontal_Inf_Tri_L: left triangular inferior frontal gyrus; Insula_R: right insula; Cingulum_Mid_L: left median cingulate gyrus; Rolandic_Oper_R: right rolandic operculum; SupraMarginal_R: right supramarginal gyrus; Supp_Motor_Area_R: right supplementary motor area; Caudate_R: right caudate nucleus; Fusiform_L: left fusiform gyrus.

**Table S14.** Correlation between aberrant dFNC in state 1 and abnormal network switching rate in patients with ARHL

| dFNC | Functional network | Network switching rate | *r* | *p* | *q_FDR_* |
| --- | --- | --- | --- | --- | --- |
| CEN(IC_30_)-VN(IC_40_) | DMN | Frontal_Sup_Medial_R | -0.248^a^ | 0.171 | 0.598 |
|  |  | Precuneus_L | 0.265^a^ | 0.143 | 0.598 |
|  |  | Precuneus_R | 0.302^a^ | 0.093 | 0.598 |
|  | CEN | Frontal_Sup_R | 0.092^b^ | 0.615 | 0.922 |
|  |  | Frontal_Mid_R | 0.261^a^ | 0.149 | 0.598 |
|  |  | Frontal_Mid_Orb_L | -0.023^a^ | 0.902 | 0.922 |
|  | SN | Frontal_Inf_Tri_L | -0.143^a^ | 0.434 | 0.922 |
|  |  | Insula_R | -0.018^a^ | 0.922 | 0.922 |
|  |  | Cingulum_Mid_L | -0.073^a^ | 0.691 | 0.922 |
|  | AUN | Rolandic_Oper_R | -0.027^a^ | 0.885 | 0.922 |
|  |  | SupraMarginal_R | 0.144^b^ | 0.432 | 0.922 |
|  | SMN | Supp_Motor_Area_R | -0.058^a^ | 0.754 | 0.922 |
|  | SCN | Caudate_R | -0.092^a^ | 0.617 | 0.922 |
|  | VN | Fusiform_L | 0.055^a^ | 0.766 | 0.922 |

Partial correlation analysis was applied for evaluating the correlation between aberrant dFNC in state 1 and abnormal network switching rate, controlled for age, gender and educational level; FDR: false discovery rate; ^a^: Pearson’s correlation; ^b^: Spearman’s correlation; dFNC, dynamic functional network connectivity; IC: independent component; DMN: default model network; SN: salience network; CEN: central executive network; VN: visual network; AUN: auditory network; SMN: sensory motor network; SCN: subcortical network; Frontal_Sup_Medial_R: right superior medial frontal gyrus; Precuneus_L: left precuneus; Precuneus_R: right precuneus; Frontal_Sup_R: right dorsolateral superior frontal gyrus; Frontal_Mid_R: right middle frontal gyrus; Frontal_Mid_Orb_L: left orbital middle frontal gyrus; Frontal_Inf_Tri_L: left triangular inferior frontal gyrus; Insula_R: right insula; Cingulum_Mid_L: left median cingulate gyrus; Rolandic_Oper_R: right rolandic operculum; SupraMarginal_R: right supramarginal gyrus; Supp_Motor_Area_R: right supplementary motor area; Caudate_R: right caudate nucleus; Fusiform_L: left fusiform gyrus.

**Table S15.** Correlation between aberrant dFNC in state 4 and abnormal network switching rate in patients with ARHL

| dFNC | Functional network | Network switching rate | *r* | *p* | *q_FDR_* |
| --- | --- | --- | --- | --- | --- |
| DMN(IC_14_)-DMN(IC_33_) | DMN | Frontal_Sup_Medial_R | 0.065^b^ | 0.622 | 0.907 |
|  |  | Precuneus_L | 0.203^b^ | 0.120 | 0.897 |
|  |  | Precuneus_R | 0.153^b^ | 0.244 | 0.897 |
|  | CEN | Frontal_Sup_R | 0.202^b^ | 0.122 | 0.897 |
|  |  | Frontal_Mid_R | 0.101^b^ | 0.444 | 0.897 |
|  |  | Frontal_Mid_Orb_L | 0.076^b^ | 0.564 | 0.907 |
|  | SN | Frontal_Inf_Tri_L | 0.121^b^ | 0.356 | 0.897 |
|  |  | Insula_R | 0.149^b^ | 0.255 | 0.897 |
|  |  | Cingulum_Mid_L | 0.173^b^ | 0.185 | 0.897 |
|  | AUN | Rolandic_Oper_R | 0.082^b^ | 0.533 | 0.907 |
|  |  | SupraMarginal_R | 0.203^b^ | 0.119 | 0.897 |
|  | SMN | Supp_Motor_Area_R | 0.062^b^ | 0.637 | 0.907 |
|  | SCN | Caudate_R | 0.039^b^ | 0.768 | 0.911 |
|  | VN | Fusiform_L | 0.106^b^ | 0.418 | 0.897 |
| SN(IC_23_)-DMN(IC_33_) | DMN | Frontal_Sup_Medial_R | 0.065^a^ | 0.623 | 0.907 |
|  |  | Precuneus_L | 0.049^a^ | 0.712 | 0.911 |
|  |  | Precuneus_R | 0.112^a^ | 0.394 | 0.897 |
|  | CEN | Frontal_Sup_R | 0.160^b^ | 0.221 | 0.897 |
|  |  | Frontal_Mid_R | 0.113^a^ | 0.388 | 0.897 |
|  |  | Frontal_Mid_Orb_L | 0.052^a^ | 0.695 | 0.911 |
|  | SN | Frontal_Inf_Tri_L | 0.172^a^ | 0.188 | 0.897 |
|  |  | Insula_R | 0.057^a^ | 0.667 | 0.911 |
|  |  | Cingulum_Mid_L | 0.134^a^ | 0.309 | 0.897 |
|  | AUN | Rolandic_Oper_R | 0.102^a^ | 0.440 | 0.897 |
|  |  | SupraMarginal_R | 0.019^b^ | 0.887 | 0.940 |
|  | SMN | Supp_Motor_Area_R | 0.372^a^ | 0.003 | 0.238 |
|  | SCN | Caudate_R | 0.129^a^ | 0.325 | 0.897 |
|  | VN | Fusiform_L | 0.014^a^ | 0.913 | 0.940 |
| DMN(IC_14_)-VN(IC_40_) | DMN | Frontal_Sup_Medial_R | 0.060^b^ | 0.648 | 0.907 |
|  |  | Precuneus_L | 0.040^b^ | 0.761 | 0.911 |
|  |  | Precuneus_R | 0.004^b^ | 0.977 | 0.991 |
|  | CEN | Frontal_Sup_R | 0.210^b^ | 0.107 | 0.897 |
|  |  | Frontal_Mid_R | 0.062^b^ | 0.635 | 0.907 |
|  |  | Frontal_Mid_Orb_L | 0.017^b^ | 0.899 | 0.940 |
|  | SN | Frontal_Inf_Tri_L | 0.133^b^ | 0.310 | 0.897 |
|  |  | Insula_R | 0.283^b^ | 0.028 | 0.897 |
|  |  | Cingulum_Mid_L | 0.068^b^ | 0.604 | 0.907 |
|  | AUN | Rolandic_Oper_R | 0.032^b^ | 0.806 | 0.930 |
|  |  | SupraMarginal_R | 0.065^b^ | 0.620 | 0.907 |
|  | SMN | Supp_Motor_Area_R | 0.050^b^ | 0.702 | 0.911 |
|  | SCN | Caudate_R | 0.221^b^ | 0.089 | 0.897 |
|  | VN | Fusiform_L | 0.137^b^ | 0.295 | 0.897 |
| VN(IC_29_)-CEN(IC_30_) | DMN | Frontal_Sup_Medial_R | 0.106^a^ | 0.419 | 0.897 |
|  |  | Precuneus_L | 0.069^a^ | 0.598 | 0.907 |
|  |  | Precuneus_R | 0.109^a^ | 0.408 | 0.897 |
|  | CEN | Frontal_Sup_R | 0.168^b^ | 0.200 | 0.897 |
|  |  | Frontal_Mid_R | 0.182^a^ | 0.165 | 0.897 |
|  |  | Frontal_Mid_Orb_L | 0.017^a^ | 0.896 | 0.940 |
|  | SN | Frontal_Inf_Tri_L | 0.075^a^ | 0.571 | 0.907 |
|  |  | Insula_R | 0.187^a^ | 0.152 | 0.897 |
|  |  | Cingulum_Mid_L | 0.073^a^ | 0.577 | 0.907 |
|  | AUN | Rolandic_Oper_R | 0.165^a^ | 0.208 | 0.897 |
|  |  | SupraMarginal_R | 0.045^b^ | 0.731 | 0.911 |
|  | SMN | Supp_Motor_Area_R | 0.168^a^ | 0.198 | 0.897 |
|  | SCN | Caudate_R | 0.145^a^ | 0.269 | 0.897 |
|  | VN | Fusiform_L | 0.083^a^ | 0.528 | 0.907 |
| VN(IC_36_)-VN(IC_40_) | DMN | Frontal_Sup_Medial_R | 0.097^a^ | 0.462 | 0.897 |
|  |  | Precuneus_L | 0.063^a^ | 0.633 | 0.907 |
|  |  | Precuneus_R | 0.108^a^ | 0.411 | 0.911 |
|  | CEN | Frontal_Sup_R | 0.017^b^ | 0.896 | 0.940 |
|  |  | Frontal_Mid_R | 0.123^a^ | 0.351 | 0.897 |
|  |  | Frontal_Mid_Orb_L | 0.117^a^ | 0.372 | 0.897 |
|  | SN | Frontal_Inf_Tri_L | 0.030^a^ | 0.820 | 0.930 |
|  |  | Insula_R | 0.119^a^ | 0.363 | 0.897 |
|  |  | Cingulum_Mid_L | 0.100^a^ | 0.449 | 0.897 |
|  | AUN | Rolandic_Oper_R | 0.016^a^ | 0.902 | 0.940 |
|  |  | SupraMarginal_R | 0.001^b^ | 0.994 | 0.994 |
|  | SMN | Supp_Motor_Area_R | 0.029^a^ | 0.823 | 0.930 |
|  | SCN | Caudate_R | 0.108^a^ | 0.411 | 0.897 |
|  | VN | Fusiform_L | 0.041^a^ | 0.756 | 0.911 |

Partial correlation analysis was applied for evaluating the correlation between dFNC in state 4 and network switching rate, controlled for age, gender and educational level; FDR: false discovery rate; ^a^: Pearson’s correlation; ^b^: Spearman’s correlation; dFNC, dynamic functional network connectivity; IC: independent component; DMN: default model network; SN: salience network; CEN: central executive network; VN: visual network; AUN: auditory network; SMN: sensory motor network; SCN: subcortical network; Frontal_Sup_Medial_R: right superior medial frontal gyrus; Precuneus_L: left precuneus; Precuneus_R: right precuneus; Frontal_Sup_R: right dorsolateral superior frontal gyrus; Frontal_Mid_R: right middle frontal gyrus; Frontal_Mid_Orb_L: left orbital middle frontal gyrus; Frontal_Inf_Tri_L: left triangular inferior frontal gyrus; Insula_R: right insula; Cingulum_Mid_L: left median cingulate gyrus; Rolandic_Oper_R: right rolandic operculum; SupraMarginal_R: right supramarginal gyrus; Supp_Motor_Area_R: right supplementary motor area; Caudate_R: right caudate nucleus; Fusiform_L: left fusiform gyrus.

**Table S16.** Cognition- and age-related subgroup analyses: between-group comparisons for aberrant static functional network connectivity in the individuals with ARHL

| Subgroup | sFNC | *t* | *p* | *q_FDR_* |
| --- | --- | --- | --- | --- |
| MoCA<26 vs. MoCA≥26 |  |  |  |  |
|  | CEN(IC_30_)-DMN(IC_33_) | -0.154 | 0.878 | 0.878 |
|  | DMN(IC_14_)-DMN(IC_33_) | 0.876 | 0.385 | 0.769 |
|  | DMN(IC_14_)-VN(IC_40_) | 0.914 | 0.364 | 0.769 |
|  | SN(IC_23_)- DMN(IC_33_) | -0.178 | 0.859 | 0.878 |
| age≥60 vs. age<60 |  |  |  |  |
|  | CEN(IC_30_)-DMN(IC_33_) | 0.789 | 0.433 | 0.578 |
|  | DMN(IC_14_)-DMN(IC_33_) | -1.305 | 0.197 | 0.393 |
|  | DMN(IC_14_)-VN(IC_40_) | 0.338 | 0.737 | 0.737 |
|  | SN(IC_23_)- DMN(IC_33_) | 2.231 | 0.029 | 0.117 |

General linear model was applied for comparing between-group comparisons for aberrant static functional network connectivity in the individuals with ARHL in subgroup analyses, controlled for age, gender and educational level (cognition-related subgroup analysis) and gender and educational level (age-related subgroup analysis), respectively; FDR: false discovery rate; ARHL: age-related hearing loss; sFNC, static functional network connectivity; IC: independent component; DMN: default model network; SN: salience network; VN: visual network; CEN: central executive network.

**Table S17.** Cognition- and age-related subgroup analyses: between-group comparisons for aberrant dynamic functional connectivity in state 4 in the individuals with ARHL

| Subgroup | dFNC in state 4 | *t* | *p* | *q_FDR_* |
| --- | --- | --- | --- | --- |
| MoCA<26 vs. MoCA≥26 |  |  |  |  |
|  | VN(IC_29_)-CEN(IC_30_) | -0.681 | 0.499 | 0.871 |
|  | DMN(IC_14_)-DMN(IC_33_) | 0.539 | 0.592 | 0.871 |
|  | DMN(IC_14_)-VN(IC_40_) | 0.163 | 0.871 | 0.871 |
|  | SN(IC_23_)-DMN(IC_33_) | -0.631 | 0.530 | 0.871 |
|  | VN(IC_36_)-VN(IC_40_) | 0.462 | 0.646 | 0.871 |
| age≥60 vs. age<60 |  |  |  |  |
|  | VN(IC_29_)-CEN(IC_30_) | 1.190 | 0.239 | 0.546 |
|  | DMN(IC_14_)-DMN(IC_33_) | -1.844 | 0.070 | 0.350 |
|  | DMN(IC_14_)-VN(IC_40_) | 0.447 | 0.656 | 0.764 |
|  | SN(IC_23_)-DMN(IC_33_) | 0.986 | 0.328 | 0.546 |
|  | VN(IC_36_)-VN(IC_40_) | 0.302 | 0.764 | 0.350 |

General linear model was applied for comparing between-group comparisons for aberrant dynamic functional network connectivity in state 4 in the individuals with ARHL in subgroup analyses, controlled for age, gender and educational level (cognition-related subgroup analysis) and gender and educational level (age-related subgroup analysis), respectively; FDR: false discovery rate; ARHL: age-related hearing loss; dFNC, dynamic functional network connectivity; IC: independent component; DMN: default model network; SN: salience network; VN: visual network; CEN: central executive network.

**Table S18.** Cognition- and age-related subgroup analyses: between-group comparisons for aberrant dynamic network metrics in the individuals with ARHL

| Subgroup | dynamic network metrics | *t* | *p* | *q_FDR_* |
| --- | --- | --- | --- | --- |
| MoCA<26 vs. MoCA≥26 |  |  |  |  |
|  | Fraction time in state 3 | -0.408 | 0.685 | - |
|  | transition of number among states | 1.767 | 0.082 | - |
| age≥60 vs. age<60 |  |  |  |  |
|  | Fraction time in state 3 | -1.690 | 0.096 | - |
|  | transition of number among states | -1.001 | 0.320 | - |

General linear model was applied for comparing between-group comparisons for aberrant static functional network connectivity in the individuals with ARHL in subgroup analyses, controlled for age, gender and educational level (cognition-related subgroup analysis) and gender and educational level (age-related subgroup analysis), respectively; FDR: false discovery rate; ARHL: age-related hearing loss.

**Table S19.** Cognition- and age-related subgroup analyses: between-group comparisons for aberrant dynamic functional connectivity variability in the individuals with ARHL

| Subgroup | dFNC variability | *t* | *p* | *q_FDR_* |
| --- | --- | --- | --- | --- |
| MoCA<26 vs. MoCA≥26 |  |  |  |  |
|  | DMN(IC_14_)-AUN(IC_38_) | 1.217 | 0.228 | 0.228 |
|  | AUN(IC_16_)-SN(IC_23_) | 1.565 | 0.123 | 0.228 |
| age≥60 vs. age<60 |  |  |  |  |
|  | DMN(IC_14_)-AUN(IC_38_) | -1.406 | 0.165 | 0.808 |
|  | AUN(IC_16_)-SN(IC_23_) | -0.244 | 0.808 | 0.808 |

General linear model was applied for comparing between-group comparisons for aberrant dynamic functional network connectivity variability in the individuals with ARHL in subgroup analyses, controlled for age, gender and educational level (cognition-related subgroup analysis) and gender and educational level (age-related subgroup analysis), respectively; FDR: false discovery rate; ARHL: age-related hearing loss; dFNC, dynamic functional network connectivity; IC: independent component; DMN: default model network; SN: salience network; AUN: auditory network.

**Table S20.** Cognition- and age-related subgroup analyses: between-group comparisons for aberrant network switching rate in the individuals with ARHL

| Functional network | Node | *t* | *p* | *q_FDR_* |
| --- | --- | --- | --- | --- |
| MoCA<26 vs. MoCA≥26 |  |  |  |  |
| Default mode network | SFGmed.R | -1.201 | 0.234 | 0.766 |
|  | PCUN.L | 0.062 | 0.951 | 0.954 |
|  | PCUN.R | 0.302 | 0.763 | 0.954 |
| Central executive network | SFGdor.R | -0.842 | 0.403 | 0.806 |
|  | MFG.R | -0.870 | 0.388 | 0.806 |
|  | ORBmid.L | -1.105 | 0.273 | 0.766 |
| Salience network | IFGtriang.L | -0.303 | 0.763 | 0.954 |
|  | INS.R | -1.108 | 0.272 | 0.766 |
|  | DCG.L | -0.687 | 0.495 | 0.866 |
| Auditory network | ROL.R | -0.515 | 0.609 | 0.947 |
|  | SMG.R | -1.633 | 0.108 | 0.766 |
| Subcortical network | CAU.R | 0.200 | 0.842 | 0.954 |
| Visual network | FFG.L | -1.130 | 0.263 | 0.766 |
| Sensorimotor network | SMA.R | -0.058 | 0.954 | 0.954 |
| age≥60 vs. age<60 |  |  |  |  |
| Default mode network | SFGmed.R1 | 1.098 | 0.277 | 0.836 |
|  | PCUN.L2 | 1.048 | 0.299 | 0.836 |
|  | PCUN.R3 | 1.065 | 0.291 | 0.836 |
| Central executive network | SFGdor.R4 | 1.872 | 0.066 | 0.836 |
|  | MFG.R5 | 0.205 | 0.838 | 0.952 |
|  | ORBmid.L6 | 1.201 | 0.234 | 0.836 |
| Salience network | IFGtriang.L7 | 0.410 | 0.683 | 0.952 |
|  | INS.R8 | 0.351 | 0.727 | 0.952 |
|  | DCG.L9 | 0.061 | 0.952 | 0.952 |
| Auditory network | ROL.R10 | 0.390 | 0.698 | 0.952 |
|  | SMG.R11 | 0.457 | 0.649 | 0.952 |
| Subcortical network | CAU.R12 | -0.140 | 0.889 | 0.952 |
| Visual network | FFG.L13 | -2.855 | 0.005 | 0.952 |
| Sensorimotor network | SMA.R14 | 0.163 | 0.871 | 0.952 |

General linear model was applied for comparing the differences of node switching rate in functional networks in the individuals with ARHL in subgroup analyses, controlled for age, gender and educational level (cognition-related subgroup analysis) and gender and educational level (age-related subgroup analysis), respectively; FDR: false discovery rate; ARHL: age-related hearing loss; ORBmid.L: left orbital middle frontal gyrus; IFGtriang.L: left triangular inferior frontal gyrus; DCG.L: median cingulate and paracingulate gyrus; PCUN.L: left precuneus; FFG.L: left fusiform gyrus; SFGmed.R: right medial superior frontal gyrus; SFGdor.R: right dorsolateral superior frontal gyrus; SMA.R: right supplementary motor area; MFG.R: right middle frontal gyrus; CAU.R: right caudate nucleus; INS.R: right insula; ROL.R: right rolandic operculum; SMG.R: right supramarginal gyrus; PCUN.R: right precuneus.

**Table S21.** The sensitivity of the cognitive performance measures for detecting cognitive related changes used in the current study

| Measures | Cut-off | *diseases* | *sensitivity* |
| --- | --- | --- | --- |
| MMSE^33^ | 24 | dementia | 0.910 |
| MoCA^33^ | 26 | mild cognitive decline | 0.890 |
| AVLT^34^ | 3 | mild cognitive impairment | 0.926 |
| TMT-A^35^ | 72.5 | mild cognitive impairment | 0.484 |
| TMT-B^36^ | 135.5 | mild cognitive impairment | 0.518 |
| DST-forward^37^ | 8 | major cognitive disorders | 0.460 |
| DST-backward^37^ | 3 | major cognitive disorders | 0.770 |
| DSST^38^ | 37 | cognitive deficit caused by ischaemic leukoaraiosis | 0.910 |
| CDT^39^ | Shulman scoring system | dementia | 0.820 |
| CFT^40^ | 28 | subjective cognitive decline | 0.880-0.929 |
| VFT^41^ | 19 | dementia | 0.800 |

MMSE, Mini Mental State Exam; MoCA, Montreal Cognitive Assessment; AVLT, Auditory Verbal Learning Test; CFT, Complex Figure Test; DST, Digit Span Test. TMT-A, Trail Making Test-Part A; TMT-B, Trail Making Test-Part B; CDT, Clock Drawing Test; VFT, Verbal Fluency Test; DSST, Digit Symbol Substitution Test;
